# Changes to insulin sensitivity in glucose clearance systems and redox following dietary supplementation with a novel cysteine-rich protein: A pilot randomized controlled trial in humans with type-2 diabetes

**DOI:** 10.1016/j.redox.2023.102918

**Published:** 2023-10-05

**Authors:** W.M. Peeters, M. Gram, G.J. Dias, M.C.M. Vissers, M.B. Hampton, N. Dickerhof, A.E. Bekhit, M.J. Black, J. Oxbøll, S. Bayer, M. Dickens, K. Vitzel, P.W. Sheard, K.M. Danielson, L.D. Hodges, J.C. Brønd, J. Bond, B.G. Perry, L. Stoner, J. Cornwall, D.S. Rowlands

**Affiliations:** a Metabolic and Microvascular Laboratory, School of Sport, Exercise and Nutrition, Massey University, Wellington, Auckland, New Zealand; b School of Biomedical, Nutritional and Sport Science, Newcastle University, United Kingdom; c Department of Anatomy, University of Otago, Dunedin, New Zealand; d Centre for Free Radical Research, Department of Pathology and Biomedical Science, University of Otago, Christchurch, New Zealand; e Department of Food Sciences, University of Otago, Dunedin, New Zealand; f Department of Sports Science and Clinical Biomechanics, Faculty of Health Sciences, University of Southern Denmark, Odense, Denmark; g Department of Exercise and Sport Science, University of North Carolina, Chapel Hill, USA; h Centre for Early Learning in Medicine, University of Otago, Dunedin, New Zealand; i School of Health Sciences, Massey University, Wellington, Auckland, New Zealand; j Department of Physiology, University of Otago, Dunedin, New Zealand; k Department of Anaesthesiology and Surgery, University of Otago, Wellington, New Zealand

**Keywords:** Keratin, whey, Type-2 diabetes, Glutathione, Peroxiredoxin, Oxidative stress

## Abstract

We recently developed a novel keratin-derived protein (KDP) rich in cysteine, glycine, and arginine, with the potential to alter tissue redox status and insulin sensitivity. The KDP was tested in 35 human adults with type-2 diabetes mellitus (T2DM) in a 14-wk randomised controlled pilot trial comprising three 2×20 g supplemental protein/day arms: KDP-whey (KDPWHE), whey (WHEY), non-protein isocaloric control (CON), with standardised exercise. Outcomes were measured morning fasted and following insulin-stimulation (80 mU/m^2^/min hyperinsulinaemic-isoglycaemic clamp). With KDPWHE supplementation there was good and very-good evidence for moderate-sized increases in insulin-stimulated glucose clearance rate (GCR; 26%; 90% confidence limits, CL 2%, 49%) and skeletal-muscle microvascular blood flow (46%; 16%, 83%), respectively, and good evidence for increased insulin-stimulated sarcoplasmic GLUT4 translocation (18%; 0%, 39%) vs CON. In contrast, WHEY did not effect GCR (-2%; -25%, 21%) and attenuated HbA1c lowering (14%; 5%, 24%) vs CON. KDPWHE effects on basal glutathione in erythrocytes and skeletal muscle were unclear, but in muscle there was very-good evidence for large increases in oxidised peroxiredoxin isoform 2 (oxiPRX2) (19%; 2.2%, 35%) and good evidence for lower GPx1 concentrations (-40%; -4.3%, -63%) vs CON; insulin stimulation, however, attenuated the basal oxiPRX2 response (4%; -16%, 24%), and increased GPx1 (39%; -5%, 101%) and SOD1 (26%; -3%, 60%) protein expression. Effects of KDPWHE on oxiPRX3 and NRF2 content, phosphorylation of capillary eNOS and insulin-signalling proteins upstream of GLUT4 translocation Akt^Ser437^ and AS160^Thr642^ were inconclusive, but there was good evidence for increased IRS^Ser312^ (41%; 3%, 95%), insulin-stimulated NFκB-DNA binding (46%; 3.4%, 105%), and basal PAK-1^Thr423^/2^Thr402^ phosphorylation (143%; 66%, 257%) vs WHEY. Our findings provide good evidence to suggest that dietary supplementation with a novel edible keratin protein in humans with T2DM may increase glucose clearance and modify skeletal-muscle tissue redox and insulin sensitivity within systems involving peroxiredoxins, antioxidant expression, and glucose uptake.

## Highlights

• Keratin derived protein blended with whey (KDPWHE) enhanced glucose clearance.
• On insulin stimulation, KDPWHE increased muscle blood flow and GLUT4 translocation.
• Increased basal oxidised PRX2 with KDPWHE was attenuated with insulin stimulation.
• Insulin sensitivity was associated with increased antioxidant expression (GPx1, SOD1).

## Graphical abstract

**Figure:**
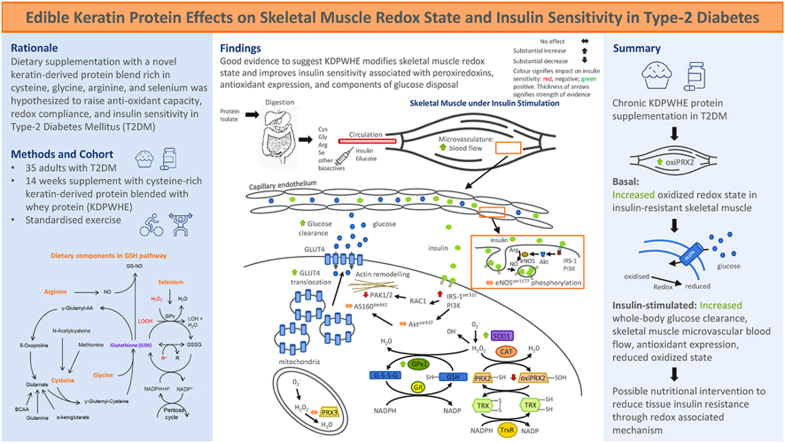

## Abbreviations:

S160: Akt substrate 160 kDa
C/F_i_: capillaries per individual fibre
CC: capillary contacts
CFPE: capillary-to-fibre-perimeter exchange
CL: confidence limits
CON: non-protein isocaloric control
COXIV: cytochrome *c* oxidase IV
CS: citrate synthase
eNOS: endothelial nitric oxide synthase
GCR: glucose clearance rate
GLUT4: glucose transporter 4
GSH: glutathione
GSSG: glutathione disulphide
GPx1: glutathione peroxidase 1
IRS: insulin receptor substrate
KDP: keratin-derived protein
KDPWHE: keratin-derived protein with whey
mBF: microvascular blood flow
mBV: microvascular vasodilation, representing tissue sectional blood volume
MVPA: moderate-to-vigorous physical activity
NEM: N-Ethyl maleimide
NF-κB: nuclear factor kappa light chain enhancer of activated B cells
NOX2: NAD(P)H oxidase
NRF2: nuclear factor erythroid 2–related factor 2
1RM: one-repetition maximum
oxiPRX2: oxidised peroxiredoxin 2
oxiPRX3: oxidised peroxiredoxin 3
PAK: p21 activated kinase
PP: peak power
SF: sharing factor
SOD1: superoxide dismutase 1
SIE: smallest important effect
SSD: smallest standardised difference
T2DM: type-2 diabetes mellitus
WHEY: whey protein isolate

## 1 Introduction

We recently developed a novel edible keratin-derived protein (KDP) containing uniquely high concentrations of cysteine, glycine, and arginine [1]; diets rich in these amino acids have recently been identified to inversely associate with mortality from cardiovascular and metabolic disease [2,3]. Cysteine and glycine are precursor substrates for the antioxidant glutathione (GSH), the synthesis and content of which is reduced in people with type-2 diabetes mellitus (T2DM) [4,5]. Perturbations in GSH metabolism are linked to disruptions to cellular redox state, metabolic function, and insulin resistance [4,6]. The GSH content in T2DM can be reversed with supplemental cysteine (N-acetylcysteine) and glycine [7], and cysteine, glycine, and selenium are established dietary supplements for boosting antioxidant capacity [8], while arginine, via nitric oxide (NO) dependent pathways, promotes vascular endothelial function [9,10].

Glutathione is part of the thiol/disulfide system centred around the selenium-containing glutathione peroxidases (GPx) and thiol reductases, which in conjunction with peroxide scavenging enzymes (e.g., catalase and superoxide dismutase, SOD) and proteins, such as the highly abundant peroxiredoxins (PRX) mediate the transfer of redox state to cell signalling and metabolism [[11], [12], [13], [14]]. Peroxiredoxins may be redox sensors [11,12] or redox messenger proteins, transmitting oxidation to subsequent target protein-cysteine residues for longer range signal transduction [6]. Overexpression of some PRX isoforms affects susceptibility to glucose intolerance and cardiovascular disease [[15], [16], [17]]; for example, PRX2 is implicated in skeletal muscle redox messaging and insulin sensitivity [18].

In a pilot study over 28-d in sedentary overweight mice, the ingestion of a 50/50 KDP/casein blend vs 100% casein lowered blood glucose (11 vs 19 mmol/L; difference 8 mmol/L, 90% confidence interval 5 to 11 mmol/L) [19], but effects of KDP in humans are unknown. Accordingly, in a proof-of-principle trial in humans with insulin resistance, we gathered evidence in support of the hypothesis that supplementation with KDP could promote a more reduced (antioxidant effect) erythrocyte and skeletal muscle redox state monitored via the oxidation status of GSH and PRX, which would associate with improved blood-glucose clearance and insulin sensitivity within measures of primary skeletal muscle nutrient delivery pathways, including microvascular blood flow [20], insulin-receptor signalling and glucose transporter 4 (GLUT4) translocation [21].

## 2 Materials and Methods

Men and women with T2DM (n = 35; see Results section for cohort descriptive statistics) completed a 14-wk placebo-controlled randomised trial comprising standardised exercise 5 d/wk and 2×20 g protein/d dietary supplementation in three arms: KDP-whey blend (KDPWHE), whey (WHEY), non-protein isocaloric control (CON). The preparation and nutritional properties of the novel KDP protein were evaluated for the first time in humans. The experiment derived outcomes from pre-post (week 0 and week 15) measures of tissue redox status and cell signalling in blood and insulin-stimulated (hyperinsulinaemic-isoglycaemic clamp) skeletal muscle.

### 2.1 Preparation, analysis and reducing potential of the keratin-derived protein (KDP) and whey protein isolate

#### 2.1.1 Preparation of KDP isolate

Double-scoured wool (Wool Services International, Christchurch, New Zealand) was solubilized and degraded for nutritional application using a novel process in which a reaction mixture was formed containing food grade citric acid (90 mM) and ascorbic acid (6 mM) at pH 2.3. Ascorbic acid acts as a stabilizing and reducing agent preventing repolymerization of keratin protein through reformation of keratin disulphide bonds. The reaction mixture was processed by microwave radiation, heat and pressure, using a temperature-controlled 20 kW, 2450 MHz magnetron microwave into a reaction mix with a pH of 4.1. The reaction mix was centrifuged to generate three separate fractions: a liquid fraction (supernatant), emulsified fraction (precipitate), and solid fraction (plug). The supernatant, containing high concentrations of soluble components: citric acid, ascorbate and free amino acids, were discarded. The high molecular-weight precipitate and plug were freeze dried, ground to a fine powder, combined (50:50 ratio), and vacuum packed for storage prior to use. The full KDP production process is described in Ref. [22].

#### 2.1.2 Proximate analysis of KDP and WHEY

Proximate analysis was carried out according to the 1990 Association of Official Analytical Chemists methods. Amino acids were analysed in triplicate with a standard hydrochloric acid hydrolysis followed by RP HPLC separation using AccQ Tag derivatization. Cysteine/methionine were analysed using performic acid oxidation (AOAC 994.12). The moisture, ash, fat, and protein contents (g/100 g) in the samples were determined as follows; the protein content was determined using Kjeldahl (AOAC Method 991.20), moisture content was determined by drying at 106 °C for 24 h (ISO R-1442) and ashing was done at 550 °C for 24 h (ISO R-936). The fat content was measured according to Soxhlet method (ISO R 1443) using a Foss Tecator AB Soxtec 2050. Carbohydrates was determined by subtracting the sum of moisture, ash, fat and protein content from 100. The caloric value was calculated as described by Karp et al. [23].

#### 2.1.3 Protein extraction and solubilization of KDP and WHEY

Dried dietary protein powder (10 mg) of KDP and WHEY were extracted overnight by rotation (Mini Labroller, Labnet International Inc) at room temperature in 1 ml of extraction buffer (8 mM urea, 0.05 M Tris, 50 mM TCEP, pH 4). Extracted protein was spun down at 3000 g for 5 min and the absorbance of the supernatant was measured at 280 nm (Nano photometer, Implen) against the appropriate blank after dilution (1:10). The protein solutions were kept at 4 °C until assessment of thiol groups.

#### 2.1.4 Reduction potential of KDP and WHEY

The accessible thiol content was determined in KDP and WHEY to assess the reduction potential. The proteins were extracted, and the diluted protein solutions were assayed by reaction with monobromobimane (MBB) and comparison to a GSH standard curve. 1 mM GSH (in 1 mM TCEP, 2.7 mM KCl, 0.14 M NaCl, 8 mM Na_2_HPO_4_, 1.5 mM KH_2_PO_4_, in PBS, pH 8) was pipetted into a black 96 well plate (Nunc-Immuno MicroWell, Sigma-Aldrich), in a range of 0 - 150 μM. The well was filled with PBS-urea buffer (1.8 M urea, 11.3 mM Tris, pH 8, total volume 195 μl). The protein solution was diluted further and 45 μl added per well and filled with PBS-urea. To start the reaction, 1 mM MBB (40 mM stock solution in acetonitrile) was added and incubated strictly in the dark for 20 min at room temperature before fluorescent detection (excitation λ = 394 nm, emission λ = 480 nm, Varioskan Flash, Thermo Fischer Scientific).

### 2.2 Human trial

#### 2.2.1 Participants

**Fig. 1:**
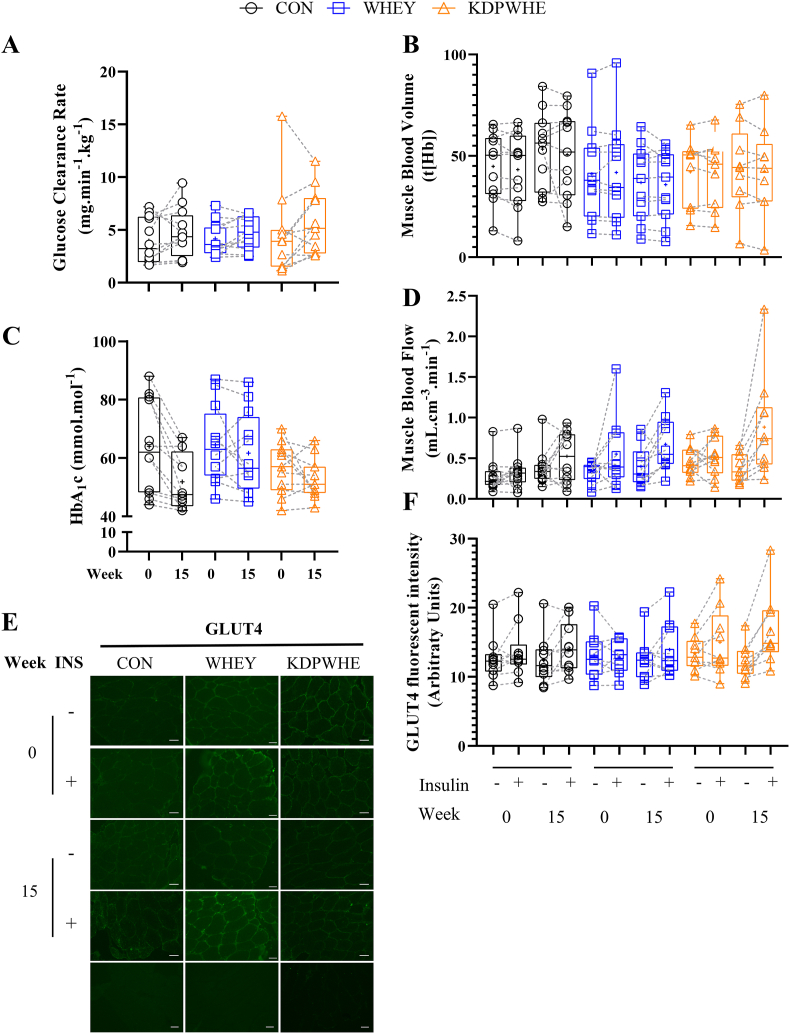
The effect of 14-weeks of CON, WHEY or KDPWHE treatment in adults with type-2 diabetes mellitus on measures of glucose clearance, nutrient delivery to tissue microvasculature and cells, and glycaemic control. (A) Glucose clearance rate (GCR) during the last 20 min of the hyperinsulinemic-isoglycaemic clamp at weeks 0 and 15. Skeletal muscle microvascular (B) blood volume (mBV), and (D) blood flow (mBF) as measured by near-infrared spectroscopy (NIRS), expressed as basal fasted (INS -) and during the last 20 min of the hyperinsulinemic-isoglycaemic clamp (INS +) at weeks 0 and 15. (C) Fasting blood [HbA1c] at weeks 0 and 15. (E) Representative immunofluorescence images of cross-sectional skeletal muscle fibres stained for glucose transporter 4 (GLUT4). GLUT4 was measured as staining intensity in the plasma membrane area (SM Fig. 3). The small grey scale lines bottom right represent 50 μm. (F) Skeletal muscle GLUT4 translocation, expressed as basal fasted (INS -) and after 60-min of insulin exposure (INS +) during the hyperinsulinemic-isoglycaemic clamp at weeks 0 and 15. Data are raw unit median, upper and lower quartiles, and range, with individual-participant data points included. Raw unit point and change score mean and SD are in SM Data 1. Outcome statistics are in Table 2. CON, non-protein isocaloric control; KDPWHE, keratin-derived protein with whey; WHEY, whey protein isolate.

Males and females of any ethnicity diagnosed with T2DM were recruited at medical centres and from the local community in Wellington (New Zealand) from January 2016 to January 2017 with final testing in May 2017. Inclusion criteria were non-insulin dependent T2DM for > one year (HbA_1_c ≥48 mmol/mol); aged 35-70 years; BMI 25-40 kg/m^2^; stable weight and without regular exercise in the past 6 months. Exclusion criteria were use of beta-blockers; moderate to severe retinopathy, nephropathy, or neuropathy; current smoker or <6 months prior, and history of cerebrovascular or cardiovascular disease. An ECG recorded during rest and a maximal cycle test were used to exclude participants with indications of ischemic heart disease or arrhythmias. Participants were provided written and oral information about risks and benefits of the trial before signing an informed consent in accordance with the Declaration of Helsinki. The study was approved by the Central Health and Disability Ethics Committee, New Zealand 14/CEN/194, and prospectively registered at the Australian New Zealand Clinical Trials Registry (ACTRN12614001197628). The full CONSORT description of recruitment and participant allocation is presented in Supplemental Material (SM) Fig. 1.

#### 2.2.2 Study design

The study was a multi-arm, double-blind, parallel group, randomized controlled trial comprising 14 wk of standardized exercise combined with dietary protein supplementation conducted in University and Hospital gyms, labs, and clinics. Participants all completed the same controlled exercise regimen and were randomized to protein treatment by an external researcher accounting for baseline glucose clearance rate (GCR), age, peak power (PP) and leg press 1RM, stratified by sex [24]. Justification for the exercise control was the therapeutic potential for exercise and dietary protein in combination (augmentation [8,25,26]) and for control of supplement delivery and timing immediately post exercise; accordingly, control of exercise likely lowered outcome variation. Another purpose was to enhance recruitment and compliance [4,26].

#### 2.2.3 Dietary protein intervention

Protein treatments were a non-protein isocaloric placebo control (CON, blend of 50% maltodextrin and 50% low protein gluten free flour); isoenergetic whey protein isolate as active control (WHEY, 40 g WPI-A895; Fonterra, New Zealand), or a blend of KDP isolate and whey protein isolate (KDPWHE, 17 g KDP+23 g whey protein isolate) (Supplementary Material, SM, Tables 1-3). The supplements were optimized for palatability, similar appearance, and packaging to achieve placebo blinding. A 70 g baked muffin prepared by a commercial bakery and 10 capsules size “0” (23 x 8 mm) in the morning and a 70 g muffin in the evening were considered most acceptable. The supplements were ingested immediately following each morning exercise session and again 1-2 h before bed. Participants were instructed to maintain pre-study dietary habits and concomitant medication throughout the intervention. 90% completion of the exercise protocol and intervention supplements were required to meet the compliance criteria. Ingestion of protein supplement after the training session was supervised. All participants reported ingesting >90% of supplement units in the eventing, where compliance was supported via receipt of automated text messages reminders in the evening to take the supplement.

#### 2.2.4 Hyperinsulinemic-isoglycaemic clamp

Participants underwent a 2-h hyperinsulinaemic-isoglycaemic clamp at weeks 0 and 15 after an overnight fast. Using dietary recall, the diet was equal the day before the clamps, and participants were provided with 250-300 ml of water in the morning prior to procedures. The post-intervention clamp was performed 46-50 h after the last exercise session to minimise influences of acute exercise induced increases in insulin sensitivity. An insulin infusion of 80 mU/m^2^/min was chosen as within the upper physiological range known to suppress hepatic gluconeogenesis in T2DM [27]. After obtaining height, weight and fat and fat-free mass from bioelectrical impedance, the participant lay supine with a cannula placed into a medial cubital vein for insulin (Actrapid; Novo Nordisk, Denmark) and glucose (25%, Baxter, Campaign) infusion using calibrated insulin (Carefusion, Alaris CC Plus, Franklin Lakes, NJ) and glucose pumps (Carefusion, Alaris GP Plus). Insulin was prepared as a 50 ml infusate using 3 ml of participant's whole blood. Insulin infusion rate was 15 ml/h. Starting glucose infusion rate (25% solution) was 0.25 mL⋅bw (kg)/h, prior to any required adjustment to maintain isoglycaemia. A second cannula in the dorsal vein of the contralateral arm was placed in a hotbox (~60 °C) to obtain arterialized blood. Blood glucose concentration was maintained isoglycaemic (at ambient [glucose]) by adjustment of the glucose infusion rate every 5 min (YSI 2300 Stat Plus, Yuba, CA). To limit urinary glucose loss, participants with high fasting [glucose] >10 mmol/L were lowered to 10 mmol/L. Glucose infusion rates were transformed to glucose clearance rates (GCR) to compare participants clamped at different ambient glucose concentration gradients [28], where the glucose infusion rate was divided by the whole blood [glucose] over the final 20 min of the clamp:

$$GCR\;\left(ml/\min/kg\right)=\frac{\text{Glucose}\;\text{infusion}\;\text{rate}\;\left(mg/\min/kg\right)}{\text{Blood}\;\text{glucose}\;\text{concentration}\;\left(mg/100ml\right)}∙100$$

A space correction was applied to the glucose infusion rate to correct non-steady state [glucose] using: (Glucose2-Glucose1)⋅0.095 [29].

#### 2.2.5 Skeletal muscle blood flow and vasodilation

Basal and insulin-stimulated skeletal muscle blood flow (mBF) and vasodilation (blood volume, mBV) were measured during the clamp using continuous-wave near infrared spectroscopy (NIRS) [30]. The mBV was used as a surrogate measure of capillary recruitment, regulated by artery-arteriolar vasodilation. The NIRS optode (PortaLite; Artinis Medical Systems BV, Elst, The Netherlands) was secured over the belly of the right *m. vastus lateralis* approximately two-thirds distance from the proximal attachment of the muscle and parallel to the orientation of the muscle fibres. Position and adipose thickness were confirmed using B-mode ultrasound (Terason; United Medical Instruments Inc., San Jose, CA). The wireless optode consisted of three light emitting diodes (LEDs), positioned 30 mm, 35 mm and 40 mm, permitting a 2.0 cm theoretical penetration distance of the signal [31]. To ensure measurements were derived from only muscle tissue, participants with an adipose tissue thickness of >2 cm were excluded from the analysis. At a rate of 10 Hz, the 3 LEDs emitted wavelengths at 760 and 850 nm to detect relative changes in the concentration of oxygenated haemoglobin [HbO_2_] and deoxygenated haemoglobin [HHb], respectively, as well as the haemoglobin concentration in the total blood volume ([tHb] = [HbO_2_] + [HHb]).

The measurements were performed before obtaining the muscle biopsies, and great care was taken to ensure that the participants lay still in a relaxed position without moving the leg while measurements were performed. mBV was calculated using the average tHb concentration (μM) over 5 min, at rest before (basal) and 120 min after insulin infusion. Using a custom and automated occlusion device, mBF was determined from the average slope of the [tHb] signal during four 15-s venous cuff occlusions (90 mmHg) with 45 s rest separating the occlusions as validated previously [30]. The signal was converted to mL/min/100 mL:

$$\text{mBF}=\frac{1}{C\times \Delta t\left[\text{Hb}\right]/\Delta t}$$

where [ΔtHb]/Δt is the average rate of tHb increase under venous occlusion (in micromoles of Hb per second) and C is the haemoglobin concentration in the blood, for which we assumed a value of 7.5 and 8.5 mmol/L for female and male participants, respectively [32].

#### 2.2.6 Skeletal muscle and erythrocyte collection and analysis

Skeletal muscle tissue (∼100 mg frozen weight) from *m. vastus lateralis* was obtained using the percutaneous Bergstrom needle technique (30) prior to insulin infusion and as close as possible to 60 min into the clamp. After applying local anaesthesia (1% Xylocaine), a small incision was made in the skin of the left leg to access the *m. vastus lateralis*. Samples were immediately freed from any visible fat and blotted dry to remove excess blood. Samples for immunofluorescence microscopy were embedded in fresh Tissue Tek OCT (Sakura Finetek, the Netherlands), immediately frozen in liquid nitrogen-cooled isopentane and stored at -80 °C until further analysis. Muscle samples for western blotting were immediately snap-frozen in liquid nitrogen and stored at -80 °C until further analysis. For GSH, GSSG, oxiPRX2 and oxiPRX3 analysis, approximately 15 mg tissue was immersed in 0.5 ml N-Ethylmaleimide (NEM) (100 mM) in PBS to prevent artefactual oxidation [12], incubated at room temperature for 5 min, snap-frozen in liquid nitrogen and stored at -80 °C until analysis. Citrate synthase (CS) and cytochrome *c* oxidase IV (COXIV) enzyme activity in skeletal muscle were determined as described [33]. GSH (as its NEM derivative) and GSSG were analysed in erythrocytes and muscle by liquid chromatography tandem mass spectrometry. Analysis of oxiPRX2 in muscle and erythrocytes, and NRF2, GPx1 and SOD1 in muscle was by Western blot. Nuclear factor kappa light chain enhancer of activated B cells (NF-κB) p50/p65 DNA-binding assay in skeletal muscle nuclear extract was of p50 and p65 combined subunits and was analysed by ELISA.

#### 2.2.7 Blood analysis

Samples for HbA_1_c analysis (BD Vacutainer® EDTA) and plasma proteins and lipids (BD Vacutainer® SST™) were analysed by Wellington Southern Community Labs, New Zealand using standard clinical chemistry techniques. Samples for analysis of insulin were stored at -80 °C until batch analysis using double antibody radioimmunoassay (Massey University, Palmerston North, New Zealand). Glucose was measured in whole blood (YSI 2300 Stat Plus). For erythrocyte GSH and PRX2, 1.5 ml of blood was immediately added to a 3 ml EDTA tube (BD Vacutainer® EDTA) prepared with 1.5 ml of 25 mg/ml of N-Ethylmaleimide (NEM, Sigma-Aldrich) in PBS. After 25 min incubation at room temperature the sample was centrifuged at 3000 g for 5 min at 4 °C. Plasma and buffy coat were removed and 100 μl of packed red cells was aliquoted and stored at -80 °C until batch analysis.

#### 2.2.8 Immunofluorescence protocol

Samples were cut at 7 μm in transverse orientation using a microtome cryostat at -20 °C (Leica CM1850, Wetzlar, Germany). Sections were mounted on superfrost microscope slides (Sigma-Aldrich, catalogue #Z692255), then air-dried for 1 h. Slides were then either stored at -80 °C, or transferred straight into 1 × TBS, pH 7.6 for initiation of a staining protocol. The following antibodies were used for visualization of insulin signalling: 1:250 anti-GLUT4 rabbit polyclonal primary antibody (Abcam, catalogue # ab654) [34], 1:500 mouse polyclonal anti-dystrophin (Sigma-Aldrich, catalogue #D8168), 1:500 rabbit polyclonal anti-p-IRS-1^Ser312^ (Invitrogen, catalogue # PA1-1054, p-IRS-1Ser307 in rodent), 1:100 rabbit polyclonal anti-eNOS (Abcam, catalogue # Ab5589), 1:100 rabbit polyclonal anti-p-eNOS^Ser1177^ (Abcam, catalogue # ab184154) or 1:100 rabbit polyclonal anti-NOX2 (Abcam, catalogue # ab80508) as primary antibodies. 1:500 Goat anti-rabbit AF488 (Alexafluor488, Invitrogen, catalogue # A-11034), 1:500 goat anti-mouse AF594 (Alexafluor594, Invitrogen, catalogue # A-11005) were used as secondary antibodies and 10 μg/mL *Ulex europaeus*-FITC conjugate (UEA-I-FITC, Sigma-Aldrich, catalogue #L9006) and 50 μg/mL wheat germ agglutinin (WGA-350, Invitrogen, catalogue #W11263) was utilized to visualize microvascular endothelial cells and sarcolemma, respectively. All antibodies were diluted in solution containing 1% BSA, 0.01% Triton-X and 0.02% Tween20.

Tissue samples were blocked for 30 min in 1% bovine serum albumin (BSA), then after washing for 3×5 min in 1 × TBS, samples were incubated in primary antibodies overnight at 4 °C. Next, samples were washed 3×5 min with 1 × TBS, 3×5 min with 2 × TBS and 3×5 min with 1 × TBS. Samples were then incubated with appropriate secondary antibodies, washed thoroughly and subsequently incubated for 1 min in TrueBlack (Lab Supply, NZ, catalogue #23007) to mask tissue autofluorescence. Finally, the samples were washed thoroughly, mounted in 50/50 TBS/glycerol and coverslipped.

#### 2.2.9 Image capture

Images of immunostained muscle fibres were captured using a Spot-RT slider (SPOT Imaging Solutions: Diagnostic Instruments, Inc., Sterling Heights, MI) cooled digital microscope camera mounted on a compound widefield fluorescence microscope (Olympus BX-50, Olympus Corporation, Tokyo, Japan) equipped with a CoolLED illuminator (CoolLED Ltd, UK). Illumination and exposure times were optimized for each antibody and kept consistent throughout the acquisition. The Alexafluor 594 was excited using Olympus U-MWIY (540-580 nm) excitation filter, the Alexafluor 488 and UEA-I-FITC were excited using Olympus U-MWIG (465-495 nm) excitation filter and the WGA-350 was excited using narrow band UV filter (Olympus U-MNU-BP, 330-385 nm). All images were taken using a 20 × oil immersion objective.

#### 2.2.10 Image analysis

Three images per sample were taken and Image J (National Institutes of Health, Bethesda, MD) was utilized for analysis. GLUT4 translocation with the plasma membrane was measured as the mean grey value within the plasma membrane region, which was determined by masking the dystrophin-positive area [34]. p-IRS-1^Ser312^, p-eNOS^Ser1177^/eNOS and NOX2 were semi-quantified by measuring the mean grey value within the plasma membrane region that was determined by masking the WGA-350-positive area and microvascular endothelial cells by masking the UEA-I-FITC-positive areas [35]. For analysing microvascular plasticity in the skeletal muscle fibres, capillaries stained with an endothelial marker (UEA-I-FITC) were counted manually. Analysis of capillary contacts (CC), capillaries per fibre on individual basis (C/F_i_), sharing factor (SF) and capillary-to-fibre-perimeter exchange (CFPE) was performed on 50 fibres per sample as has been used and validated before [36].

#### 2.2.11 Preparation of skeletal muscle tissue samples for biochemical and Western blot analyses

For GSH/GSSG and oxiPRX2/3, muscle tissue proteins were extracted as adapted from Kumar et al. [37]. Each frozen sample was placed in NP-40 lysis buffer (50 mM Tris, 137 mM NaCl, 10% glycerol, 1% NP-40, 2 mM EDTA, 100 mM NEM, pH 8) and complete protease inhibitor cocktail at 200 μl per 5 mg of tissue. The tissue was homogenized using a ground glass hand homogenizer and then left on a rotator for 2 h at 4 °C. After centrifugation at 12,000×*g* for 20 min at 4 °C, the supernatant was collected, and protein content was determined (DC Protein Assay, Bio-Rad). Samples were stored in aliquots at -80 °C for SDS-electrophoresis and GSH/GSSG analysis. For GSH/GSSG analysis, tissue extracts were treated with 4 vol of ice-cold ethanol and supernatants were analysed.

For Western blot analyses of NRF2, GPx1, SOD1, Akt, AS160, and PAK1/2, 30 mg of snap-frozen muscle tissue was homogenized in ice cold RIPA buffer (15 mM Tris, 167 mM NaCl, 0.5% sodium, deoxycholate, 0.1% SDS, 1% Triton X-100), containing complete protease and phosphatase inhibitor cocktail (at 25 μl per mg tissue, using an automatic homogenization blender (IKA) for 1 min. Sample lysates were placed in an orbital shaker for 1 h at 4 °C before centrifuging at 600×*g* for 15 min at 4 °C. Next, the supernatant was used to determine protein concentration in triplicate using a commercially available bicinchoninic acid procedure (Pierce BCA Protein Assay Kit, 23227, Invitrogen).

#### 2.2.12 Preparation of erythrocytes for GSH/GSSG analysis and SDS-electrophoresis

Frozen packed erythrocytes treated with NEM, were lysed with 270 μl deionized water, vortexed and split into three parts for further analysis. For analysis of GSH/GSSG, 200 μl of lysate was mixed with 300 μl of precipitation solution to denature the proteins (5.37 mM EDTA, 5.13 M NaCl, and 0.17 M orthophosphoric acid). After 5 min incubation at room temperature, the samples were centrifuged at 12,000 g for 10 min, and supernatants were collected for further analysis. For gel electrophoresis, 100 μl lysate were diluted further with 100 μl deionized water, and half of this dilution was added to 200 μl 3x non-reducing sample buffer, before diluting it by an additional factor of 50 with 1x sample buffer (6.6 mM Tris, 93 μM bromophenol blue, 675 mM glycerol and 0.5% SDS, pH 6.8). To calculate haemoglobin content, 5 μl of the diluted lysate was added to 1 ml Drabkin's reagent (2 mM KCN, 1 mM K_3_Fe(CN)_6_ and 12 mM NaHCO_3_), incubated at room temperature for 30 min and measured spectrophotometrically at A_540_.

#### 2.2.13 GSH/GSSG analysis

GSH (as its NEM derivative) and GSSG were analysed by liquid chromatography tandem mass spectrometry as adapted from Harwood et al. [38]. Briefly, samples were diluted with deionized water containing 0.25% formic acid (v/v) and isotopically labelled internal GSH-NEM standard was added. Analytes were separated on a Hypercarb column operated at 60 °C (Ultimate 3000 RS) with elution solvents as described [38]. The HPLC was coupled inline to an electrospray ionisation source of a mass spectrometer (Applied Biosystems 4000 QTrap). Quantification of GSH-NEM was by multiple reaction monitoring in positive ion mode. Settings for GSH-NEM were *m*/*z* 433→304 (parent ion→fragment ion) and *m/z* 436→307 for the endogenous and the isotopically labelled internal standard, respectively. Settings for GSSG were *m*/*z* 613→484 and *m/z* 619→490 for the endogenous and for the isotopically labelled internal standard, respectively. In approximately, half of the samples, GSH was lost in an accidental discard of tissue supernatant during early preparation step, resulting in loss of too many samples for insulin-stimulated analysis (requires full set of 4 biopsies) with only n = 5–6 available per group for basal GSH analysis. GSSG levels in human skeletal muscle were below the limit of detection (mean 0.13 μmol/L, SD 0.30; 0.2-1.2% of GSH) and hence the GSH/GSSG ratios were not available as an oxidative stress parameter within skeletal muscle.

#### 2.2.14 Western blot protocols

Protein extracts from skeletal muscle (4 μg for PRX2/3) and erythrocytes (6 μg for PRX2) were separated by SDS-PAGE under non-reducing conditions. Extracts from skeletal muscle (30 μg for NRF2, GPx1, SOD1 or 40 μg for Akt, AS160, and PAK1/2) were separated by SDS-PAGE under reducing conditions. Separated proteins were transferred to PVDF membranes, stained and imaged for total protein using Ponceau S (Sigma-Aldrich, Cat. P3504) prior to blocking for 1h in Tris-Buffered Saline/0.1%Tween-20 (TBS-T) containing 3% BSA. Blocked membranes were then incubated overnight at 4 °C with the following antibodies diluted in TBS-T/3%BSA; rabbit polyclonal anti-human PRX2 at 1:10,000 (R8656, Sigma-Aldrich, RRID: AB_1841096), rabbit polyclonal anti-human PRX3 at 1:10,000 (PA0030 AbFrontier, RRID: AB_1620980), rabbit monoclonal anti-NRF2 at 1:1, 000 (ab62352, Abcam, RRID:AB_944418), rabbit polyclonal anti-GPx1 at 1:1000 (11797, ThermoFisher, RRID: AB_2723218), mouse monoclonal anti-SOD1 at 1:1000 (MA1-105, ThermoFisher, RRID: AB_2536811), rabbit polyclonal anti-phospho-AS160^Thr642^ at 1:1000 (4288, Cell Signalling Technology), rabbit polyclonal anti-phospho-PAK1^Thr423^/p-PAK2^Thr402^ at 1:1000 (2601, Cell Signalling Technology), rabbit polyclonal anti-phospho-Akt^Ser437^ at 1:1000 (44-621G, ThermoFisher), rabbit monoclonal anti-Pan-Akt at 1:1000 (MA5-14916, Thermofisher), mouse monoclonal anti-α-tubulin HRP-conjugate at 1:1000 (ab40742, Abcam). PRX2/3 antibodies were characterised previously [39,40]. Other antibodies were characterised as detailed on the supplier websites. Membranes were washed 3×5 min with TBS-T before incubation with either anti-rabbit or anti-mouse HRP-conjugated secondary antibody (Sigma-Aldrich) as previously reported [39,41,42]. Membranes were again washed 3×5 min with TBS-T before visualization using enhanced chemiluminescence and the Chemidoc XRS or MD gel documentation system (Bio-Rad). After imaging, some membranes were stripped (62.5 mM Tris-HCl, 2% SDS, 0.8% β-mercaptoethanol, pH 6.8, 45 min at 50 °C), washed and reprobed for the detection of other proteins. Band intensities were quantified using ImageJ software (National Institutes of Health, MD, U.S.) and Imagelab 6.1 (Biorad). Band intensities were normalised to either an a-tubulin loading control (e.g., NRF2) or total protein as determined by Ponceau S imaging (e.g., GPx1 and SOD1). For PRX2/3 percentages of oxidised dimer in relation to the sum of oxidised dimer and reduced monomer were calculated.

#### 2.2.15 Nuclear factor kappa light chain enhancer of activated B cells (NF-κB) p50/p65 DNA-binding assay in skeletal muscle nuclear extract

The nuclear fraction was isolated using a Nuclear Extraction kit (ab113474, Abcam) and the DNA-binding activity of p65 and p50 NF-κB subunits in the lysates was evaluated by colorimetric enzyme-linked immunosorbent assay (ELISA) using the NF-κB Transcription Factor Assay kit (ab207216, Abcam), according to the manufacturer's instructions. DNA-binding activity of combined p50/p65 was expressed as intensity based on absorbance measurement at 450 nm.

#### 2.2.16 Body composition, cardiometabolic risk factors, physical performance, daily physical activity level and gastrointestinal symptoms questionnaire

Fat and fat-free mass were measured using bioelectrical impedance (Bodystat 1500 analyzer, UK) on the morning of the hyperinsulinaemic-isoglycaemic clamp. Waist circumference was measured at baseline and again prior to the final exercise session according to the International Diabetes Federation. Systolic and diastolic blood pressure was measured at baseline and again prior to the final exercise session using a Pulsecor Cardioscope (Uscom, Sydney Australia). Physical performance (cycling PP and lift 1-RM) were measured to calibrate weekly exercise load progression to 2% and 1.5%, respectively. Gastrointestinal symptoms were measured using 0-15 cm for abdominal or epigastric discomfort, or a 7-point Likert scale for nausea, belching, flatulence, and diarrhoea at the end of study week 1 and 14 [43].

#### 2.2.17 Exercise procedures

In the morning after an overnight fast and rest day, peak power (PP) was established using cycle ergometry (Velotron, WA) with resistance increasing 1 W/3 s until exhaustion [26]. The tests were repeated single blind in weeks -1, 0, 3, 6, 10 and 15. Estimated 1-repetition maximum (1RM) in bench press, leg press, lateral pull-down and hip thruster were performed in weeks -1, 0, 3 and 15 using the Brzycki multiple repetition testing procedure [44]. To control exercise as a confounder, participants engaged in 14 weeks of supervised controlled exercise training comprising four endurance and one resistance training sessions per week. All training sessions were executed fasted, and in the morning. The training started with a 2-week lead-in protocol (5-10% points lower in duration and intensity, respectively), prior to clamping exercise workload against the retest outcomes in week 3. Thereafter, weekly training load progressed 2% and 1.5% of cycling peak power and 1 RM, respectively. Endurance training was performed on the calibrated cycle ergometer. All sessions started and ended with a 3 min warmup and cool down period. Cycling on Tuesdays and Fridays consisted of 3 x 10 min (week 3-6) increasing to 3 x 12 min (week 7-14) at 60% PP with 1 min active recovery (total time 38 min–44 min by week 7). Cycling on Thursdays consisted of 5 min at 55% PP, followed by 10 x 1 min at 90% PP with 2 min active recovery. Cycling on Mondays consisted of 10 min at 55% PP, followed by 1-2-3-2-1 min at 75% PP with 1-2-3-2-one min active recovery (total time 34 min). Resistance training on Wednesdays consisted of a short exercise-specific warmup (8 repetitions at 20% of 1RM) followed by 3 sets of 10 repetitions increasing to 12 repetitions at week 7 at 55% 1RM, with 2 min rest between sets of bench press, leg press, lateral pulldown, and hip thrusters. To finish the resistance exercise, participants completed 2 x 300 m high-intensity rowing (Concept II) with 2 min rest in between.

### 2.3 Statistical analysis

#### 2.3.1 Sample size

Sample size was calculated [45] based upon the glucose disposal rate (CV 3.7%) [29] and the smallest important effect (SIE) size under the superiority and equivalence framework [[46], [47], [48], [49]] where there is a 95% probability of rejecting the non-superiority hypothesis (i.e. opposite direction) for a realistic expected magnitude of the effect [49], where a borderline small-moderate standardised true effect size is 3x the SIE [50]. The value for the SIE was chosen as 5.4% based on the relationship of change in glucose clearance rate after 12-weeks on metformin [51]. Baseline between-subject SD of 11.2% [51] was included in the estimation of sample size because the analysis included adjustment for baseline. These estimates provided a sample size of 12/group and power for a clinical superiority outcome likely compatible with the anticipated effect size of 21.5% based on GCR response vs standard pioglitazone + metformin intervention [51].

#### 2.3.2 General statistical method

Data were analysed using mixed model longitudinal analysis of covariance for change scores adjusted for the baseline value [52] using Proc Mixed (SAS Enterprise Guide 8.2.1, Cary, NC). Continuous data were analysed after 100 times the natural-log transformation to establish uniformity of error across the linear range and to express outcomes in percent. Data naturally expressed in percent (peroxiredoxin) were analysed without log-transformation. All post-pre intervention change scores were baseline (covariate) adjusted. The full analysis dataset (included dropouts) was used to evaluate the gastrointestinal symptoms questionnaire, while the per protocol dataset was used for the remaining assessments. Random effects were subject and protein treatment identity (to allow for within the model different between-subject variability in those assigned to one of the two protein conditions). Linear regression was used to estimate associations between *a priori* mechanistic modifiers oxiPRX2, GPx1, and SOD1 on GCR (GSH was excluded due to analysis problems); in these models the slope of the modifier was interacted with treatment and added to the primary model for the dependent adjusted for the baseline covariate.

Thresholds for interpreting the magnitude of effect sizes (relative to clinical thresholds or the standardized difference/Glass's *g*) are shown in Table 1 footnote. Precision of estimation is expressed as 90% confidence limits (CL), as favoured by Rothman [53], and interpreted consistent with an alpha of 0.05 (maximum error rate of 5%) for rejection of two one-sided substantial (as in superiority testing) hypotheses, relative to defined smallest important effect (SIE), be that positive/higher/increasing or negative/lower/decreasing. The approach sits within the inferential family of equivalence, non-inferiority and minimal effects or superiority testing [54,55]. Specifically, if the lower and upper limits of the 90% CL for a mean effect was substantial (>SIE) and of opposite direction, the effect was deemed unclear (failure to reject a substantial hypothesis); otherwise, the effect was deemed to have adequate precision at the 90% level [53,56,57]. The extent of overlap of the confidence interval with substantial (i.e., slight, small, moderate, large, very large, exceptionally large; see Table 1 footer) values representing the alternative hypothesis was used to assess the strength of evidence for or against the magnitude of the effect. The extent of overlap was estimated as the area of the sampling *t*-distribution falling in substantial and slight magnitudes [58,59]. Spreadsheets containing the full analysis statistics are provided in Supplementary Material (SM) Data 1.

**Table 1:** Baseline characteristics of the participants who completed the clinical trial (per protocol dataset).

	Treatment group		
	CON (n = 12)	WHEY (n = 12)	KDPWHE (n = 11)
Age, years	58 (8); range (45 to 69)	55 (8); range (39 to 66)	57 (8); range (46 to 67)
Sex, n female, %	3 (25)	5 (42)	3 (27)
Ethnicity, n number of participants			
European	6	8	6
Polynesian	3	3	2
South Asian	3	1	3
Peak Power, W	169 (45)	170 (55)	164 (38)
Leg press 1RM, kg	191 (72)	184 (81)	203 (52)
GCR, ml∙min^-1^⋅kg^-1^	4.0 (2.1)	4.2 (1.6)	4.5 (4.3)
Weight, kg	97 (14)	94 (21)	93 (26)
Height, cm	174 (10)	172 (10)	172 (7)
BMI, kg∙m^2^	32 (5)	32 (6)	33 (8)
Waist, cm	108 (11)	108 (15)	111 (18)
Hip, cm	110.5 (10.0)	113.5 (12.1)	110.9 (12.5)
Diabetes duration, years	6 (4)	8 (6)	7 (6)
Medications, n participants			
Metformin	8	8	7
Apo Cilazapril	1	0	1
Hydrochlorothiazide	0	0	1
Glucozide	4	3	3
Accupril	0	0	1
Cartia (Asprin)	1	1	1
Atorvastatin	3	1	2
Seratide	1	0	0
Flexanese	1	0	0
Exetamide	1	0	0
Losartan	0	1	1
Salazapril	0	2	0
Simvastatin	1	1	0
No Medication	3	1	2

All values are raw mean (SD). BMI, body mass index; CON, non-protein isocaloric control; GCR, glucose clearance rate; KDPWHE, keratin-derived protein with whey; WHEY, whey protein isolate.

## 3 Results

### 3.1 Protein and supplement composition

The macronutrient and amino acid composition of the KDP and WHEY protein isolates, and supplement composition are in SM Tables 1-3 Relative to the WHEY isolate, KDP was 3.5-, 3.7-, and 2.6-fold higher in cysteine/cystine, arginine, and glycine, respectively (SM Table 1). WHEY was 100% soluble, whereas the KDP fractions were largely insoluble, reducing the likelihood that free cysteine residues underwent oxidation. The accessible thiol concentration of 10 mg protein/ml solution was 3.9 mM for both KDP and WHEY. KDP contained notable selenium (0.090 mg/kg), zinc (6.34 mg/kg), and non-toxic trace heavy metals (SM Table 4).

### 3.2 Participant disposition, gastrointestinal symptoms, and supplement acceptability

Of the 43 randomized participants, 35 completed (8 dropouts, 19%) (see Consort flow diagram Figure SM1 and Table 1). Five participants experienced gastrointestinal distress or issues contributing to dropout in 3 (SM Table 5). Otherwise, the supplements were acceptable. Body composition and blood pressures, and physical performance measures were unaffected by treatment (trivial standardized differences, data not shown).

### 3.3 Glucose clearance, nutrient delivery, and sarcoplasmic GLUT4 translocation

In response to insulin stimulation (hyperinsulinaemic-isoglycaemic clamp), there was good and very good evidence for substantial (moderate standardized effect size) increases in whole-body glucose clearance rate (GCR) and skeletal muscle microvascular blood flow (mBF), respectively, and for good evidence for moderate increases in GLUT4 translocation with KDPWHE supplementation vs CON, and vs WHEY (Fig. 1; Table 2; full statistical analysis in SM1), but vasodilation (mBV), fasting blood insulin or glucose concentrations (not shown) were unaffected. In contrast, WHEY did not clearly affect GCR and there was good evidence for blood [HbA_1_c] to remain elevated relative to a decrease with CON. In contrast, blood [HbA_1_c] clearly decreased in CON and KDPWHE, with some evidence for the decrease in CON to be more than with KDPWHE, and for KDPWHE to be more than with WHEY (Table 2).

**Table 2:** The effect of 14-weeks of KDPWHE, WHEY, or CON supplementation in adults with type-2 diabetes mellitus on whole-body and skeletal-muscle glucoregulatory phenotype responses: haemoglobin A1c, basal and insulin-stimulated glucose clearance rate and, nutrient delivery to skeletal muscle microvasculature, and sarcoplasmic membrane GLUT4 content and translocation.

Parameter, condition	Within-Group Baseline-Adjusted Change^a^		Baseline-Adjusted Treatment-Group Contrast Effect Size Statistics^c^			
	Treatment (n)	Week 15-0 Change in % (90%CL)^b^	Treatment Contrast	Estimate in % (90%CL)^c^	Standardized difference (90%CL)^d^	Magnitude^d^
*Glucose clearance and glycaemic control*						
Glucose clearance rate, insulin stimulated	CON (12)	20 (3, 41)	WHEY-CON	-2 (-22, 23)	-0.41 (4.1, -4.3)	Unclear
	WHEY (12)	18 (0.4, 39)	KDPWHE-CON	24 (2, 55)	4.4 (0.3, 10.4)	Slight to very large
	KDPWHE (11)	49 (26, 75)	KDPWHE-WHEY	26 (0, 59)	4.9 (0.0, 11.1)	Slight to very large
[HbA1c], basal	CON (12)	-18 (-23, -12)	WHEY-CON	16 (-5.4, 28)	2.0 (0.7, 3.4)	Slight to moderate
	WHEY (12)	-4.2 (-12, 3.9)	KDPWHE-CON	5.8 (-4.1, 17)	0.7 (-0.5, 2.0)	Slight to small
	KDPWHE (11)	-13 (-20, -5.5)	KDPWHE-WHEY	-9.1 (-19, 1.5)	-1.1 (-2.2, 0.2)	Slight to small
*Skeletal muscle microvascular nutrient delivery and sarcoplasmic GLUT4 translocation*						
mBF, insulin stimulated	CON (12)	39 (28, 52)	WHEY-CON	-3 (-30, 33)	-0.07 (-0.82, 0.66)	Unclear
	WHEY (10)	35 (-1.5, 84)	KDPWHE-CON	46 (16, 83)	0.87 (0.35, 1.39)	Slight to large
	KDPWHE (9)	103 (63, 152)	KDPWHE-WHEY	51 (5, 116)	0.94 (0.11, 1.77)	Slight to large
mBV, insulin stimulated	CON (10)	2.2 (-0.0, 4.6)	WHEY-CON	-3 (-9, 3)	-0.08 (-0.23, 0.06)	Trivial
	WHEY (11)	-1.2 (-6.7, 4.7)	KDPWHE-CON	-3 (-10, 4)	-0.08 (-0.25, 0.09)	Unclear
	KDPWHE (8)	-1.2 (-7.7, 5.9)	KDPWHE-WHEY	0 (-8, 9)	0.00 (-0.20, 0.20)	Trivial
GLUT4, basal	CON (10)	-3.7 (-15, 8.0)	WHEY-CON	-1 (-17, 18)	-0.05 (-1.00, 0.90)	Unclear
	WHEY (9)	-4.6 (-18, 9.0)	KDPWHE-CON	-5 (-16, 9)	-0.29 (-1.02, 0.45)	Unclear
	KDPWHE (9)	-9.1 (-17, -1.6)	KDPWHE-WHEY	-4 (-18, 12)	-0.24 (-1.06, 0.59)	Unclear
GLUT4, insulin stimulated	CON (10)	19 (2.5, 36)	WHEY-CON	3 (-12, 20)	0.13 (-0.70, 0.97)	Unclear
	WHEY (9)	10.1 (-0.6, 21)	KDPWHE-CON	18 (0, 39)	0.87 (-0.03, 1.76)	Slight to large
	KDPWHE (9)	25 (13, 37)	KDPWHE-WHEY	15 (-1, 34)	0.73 (-0.07, 1.53)	Slight to large

a Refer to Fig. 1 for plots of raw unit point data and distribution statistics, SM Data 1 for detailed statistics, raw measurement units, raw unit point and change-score mean and SD.

b Data are least-squares mean Week 15-0 change score in percent with 90% confidence limits (90%CL) for single point observations or insulin-stimulated values, where the latter is the insulin-stimulated (measured during isoglycaemic clamp) minus baseline (measured pre-clamp) difference score. Data are baseline-covariate adjusted values.

c Effect statistics are baseline-adjusted estimates of the treatment effects on the week 15-0 change score with 90%CL expressed as percent.

d Magnitude of effect estimates were interpreted from the lower and upper CLs, consistent with an alpha of 0.05 (maximum error rate of 5%) for rejection of substantial (superiority) hypotheses, referenced against the smallest effect threshold. The thresholds for interpreting effect magnitudes are based on the modified Cohen *d* scale, where effects <0.2SD are slight (or trivial if the 90%CL sits within thresholds for small negative and small positive), >0.2SD are small, >0.6SD moderate, >1.2SD large, >2.0SD very large, and >4.0SD exceptional. Effect magnitudes for the two clinically-defined variables (GCR, HbA1c) are factors of magnitude relative to the smallest important clinical effect size (SIE) (GCR, 5.4% [51]; HbA1c, 5.5 mmol HbA1c per mole of total haemoglobin which translates to 8.7% of the pooled baseline HbA1c concentration 63.1 mmol/mol). The clinical factors use the same scale of effect size qualifier, but the value for the SIE replaces the Cohen *d* threshold for small differences, with the threshold for small being an effect size/SIE>1.0, with larger effect sizes increasing in step magnitude by factors of the SIE of x3 (moderate), x6 (large), x10 (very large), and x20 (exceptional), respectively [50,57].Further details about our approach to evaluating sampling uncertainty is described in the Materials and Methods section.CON, non-protein isocaloric control; KDPWHE, keratin-derived protein with whey protein blend; WHEY, whey protein isolate; microvascular blood flow (mBF); vasodilation (blood volume, mBV); glucose transporter 4 (GLUT4). Insulin stimulated is the insulin minus baseline score difference.

### 3.4 Redox environment in skeletal muscle and erythrocytes

**Fig. 2:**
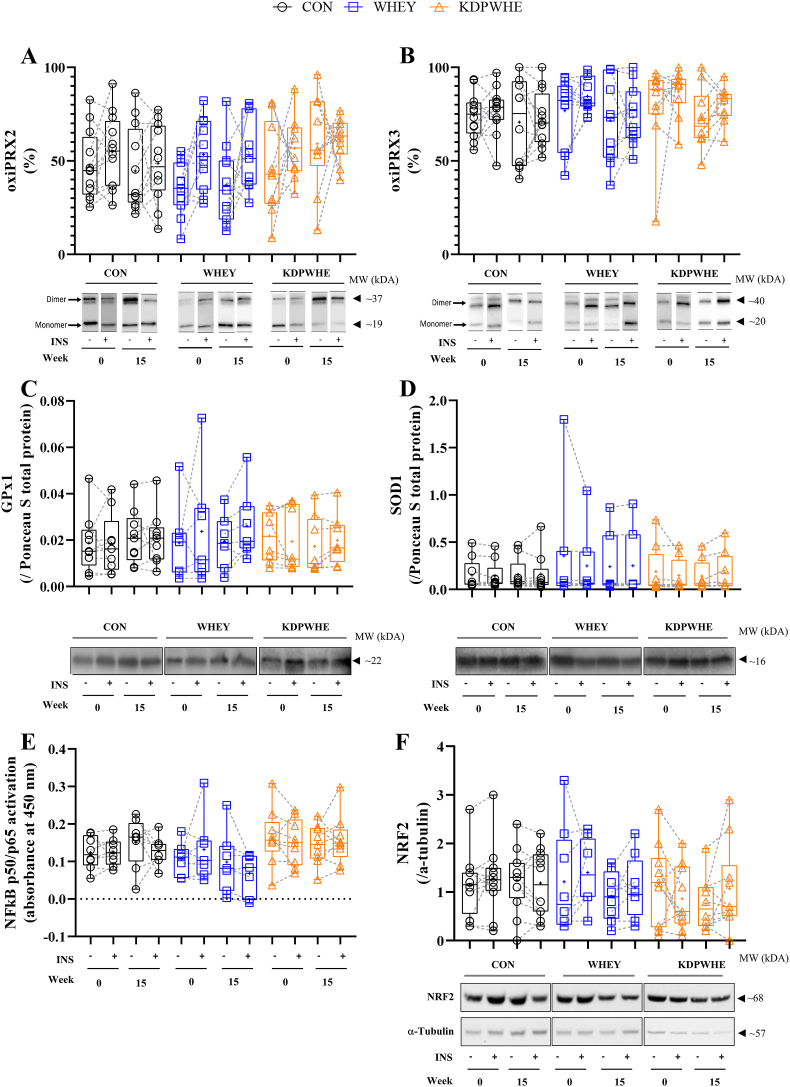
The effect of 14-weeks of CON, WHEY or KDPWHE treatment in adults with type-2 diabetes mellitus on measures of skeletal muscle redox state in responses to basal (INS -) and insulin-stimulated conditions (hyperinsulinemic-isoglycaemic; INS +), with data shown as relative protein abundance at week 0 and 15, and representative Western blots. (A) Oxidised peroxiredoxin 2 (oxiPRX2). (B) Oxidised peroxiredoxin 3 (oxiPRX3). For PRX2 and PRX3, the % oxidised was determined from the ratio of the dimer upper band at 44 kD, compared to the PRX monomer at ∼20 kD (lower band). In PRX2, bands from matched individuals at week 0 and at week 15 ± INS. (C) Glutathione peroxidase 1 (GPx1). (D) Superoxide dismutase 1 (SOD1). SOD1 and GPx1 were normalised to total protein (Ponceau S). (E) Nuclear factor kappa light chain enhancer of activated B cells (NFκB) p50/p65 DNA binding activity (activation), which was determined by ELISA. (F) Nuclear factor erythroid 2–related factor 2 (NRF2). NRF2 was normalised to tubulin. Data are raw unit median, upper and lower quartiles, and range, with individual-participant data points included. Raw unit point and change score mean and SD are in SM Data 1. Outcome statistics are in Table 3. CON, non-protein isocaloric control; KDPWHE, keratin-derived protein with whey; WHEY, whey protein isolate.

In skeletal muscle, assay error precluded firm conclusions on [GSH]. Redox status within the erythrocyte was largely unaffected by treatment (Table 3). On the other hand, more robust information on redox was drawn from skeletal muscle PRX2 (cytosolic) and PRX3 (mitochondrial), 35-50% and >70% of which was oxidised (oxiPRX2 and oxiPRX3, respectively) at baseline (Fig. 2A-B, Table 3). By week 15, there was very good evidence for oxiPRX2 to increase (19%) with KDPWHE vs CON (standardised difference, ES: large) and was 24% (ES: very large) more oxidised vs WHEY. Basal oxiPRX3 was not clearly affected by treatment, but there was good evidence for moderately lower GPx1 protein concentration by 38% and 35% with KDPWHE vs CON and vs WHEY, respectively.

**Table 3:** The effect of 14-weeks of KDPWHE, WHEY, or CON treatment in adults with type-2 diabetes mellitus on mechanistic parameters relating to skeletal-muscle tissue and erythrocyte redox environment, and muscle endogenous antioxidant enzyme content and mitochondrial enzyme plasticity.

Parameter, state	Within-Group Baseline-Adjusted Change^a^		Baseline-Adjusted Treatment-Group Contrast Effect Size Statistics^3^			
	Treatment (n)	Week 15-0 Change in % (90%CL)^b^	Treatment Contrast	Estimate % (90%CL)^b^	Standardized difference (90%CL)^3,4^	Magnitude^4^
*Skeletal muscle (V. Lateralis)*						
[GSH], basal	CON (6)	8.3 (-10.1, 30)	WHEY-CON	-28 (-96, 1143)	-1.3 (-12.2, 9.7)	Unclear
	WHEY (6)	-21, (-49, 21)	KDPWHE-CON	6.1 (-55, 151)	0.23 (-3.1 3.5)	Unclear
	KDPWHE (5)	-30 (-52, 2.3)	KDPWHE-WHEY	47 (-81, 1044)	1.5 (-6.4, 9.4)	Unclear
oxiPRX2, basal	CON (11)	-0.5 (-12, 10.9)	WHEY-CON	-5.2 (-23, 12)	-0.57 (-2.8, 1.3)	Unclear
	WHEY (11)	-5.7 (-20, 8.4)	KDPWHE-CON	19 (2.2, 35)	1.8 (0.24, 3.2)	Small to very large
	KDPWHE (11)	18.1 (6.4, 30)	KDPWHE-WHEY	24 (6.1, 41)	2.3 (0.64, 3.7)	Moderate to very large
oxiPRX2, insulin stimulated	CON (12)	-10.5 (-23, 1.6)	WHEY-CON	22 (4.2, 39)	1.4 (0.29, 2.3)	Small to very large
	WHEY (11)	-8.9 (-20, 1.9)	KDPWHE-CON	4.0 (-16, 24)	0.28 (-1.2, 1.5)	Unclear
	KDPWHE (10)	-2.3 (-7.8, 3.2)	KDPWHE-WHEY	-18 (-38, 2.9)	-1.4 (-3.4, 0.20)	Slight to very large
oxiPRX3, basal	CON (12)	-3.1 (-12, 5.8)	WHEY-CON	-2.3 (-17, 13)	-0.20 (-1.6, 1.0)	Unclear
	WHEY (10)	-5.4 (-18, 7.1)	KDPWHE-CON	0.9 (-12, 14)	0.08 (-1.1, 1.1)	Unclear
	KDPWHE (10)	-2.2 (-11.3, 6.9)	KDPWHE-WHEY	3.2 (-12, 18)	0.27 (-1.1, 1.5)	Unclear
oxiPRX3, insulin stimulated	CON (12)	-10.5 (-23, 1.6)	WHEY-CON	-1.7 (-15, 18)	0.10 (-0.91, 0.95)	Unclear
	WHEY (10)	-8.9 (-20, 1.9)	KDPWHE-CON	6.0 (-7.6, 19)	0.33 (-0.46, 1.0)	Unclear
	KDPWHE (10)	-4.6 (-11.4, 2.2)	KDPWHE-WHEY	4.3 (-8.0, 17)	0.40 (-0.49, 0.89)	Unclear
[NRF2], basal	CON (12)	1.5 (-41, 44)	WHEY-CON	-10.6 (-65, 44)	-0.18 (-1.66, 0.58)	Unclear
	WHEY (9)	5.0 (-28, 38)	KDPWHE-CON	-20 (-73, 34)	-0.35 (-2.11, 0.47)	Unclear
	KDPWHE (10)	3.7 (-53, 60)	KDPWHE-WHEY	-30 (-91, 31	-0.57 (-3.87, 0.43)	Unclear
[NRF2], insulin stimulated	CON (12)	1.5 (-41, 44)	WHEY-CON	3.5 (-51, 58)	0.05 (-1.11, 0.72)	Unclear
	WHEY (10)	5.0 (-28, 38)	KDPWHE-CON	-1.3 (-67, 65)	-0.02 (-1.75, 0.78)	Unclear
	KDPWHE (10)	3.7 (-53, 61)	KDPWHE-WHEY	2.2 (-69, 73)	0.03 (-1.84, 0.87)	Unclear
[GPx1], basal	CON (9)	20 (-8.7, 57)	WHEY-CON	-5.0 (-37, 44)	-0.09 (-0.85, 0.66)	Unclear
	WHEY (7)	14 (-17, 56)	KDPWHE-CON	-38 (-59, -6.7)	-0.87 (-1.62, -0.13)	Slight to large
	KDPWHE (8)	-26 (-45, 0.8)	KDPWHE-WHEY	-35 (-58, 1.2)	-0.78 (-1.58, 0.02)	Slight to large
[GPx1], insulin stimulated	CON (9)	1.3 (-20, 29)	WHEY-CON	42 (-1.7, 106)	0.81 (-0.04, 1.67)	Slight to large
	WHEY (7)	44 (8.8, 91)	KDPWHE-CON	39 (-4.5, 101)	0.76 (-0.11, 1.62)	Slight to large
	KDPWHE (8)	41 (5.6, 87)	KDPWHE-WHEY	-2.5 (-35, 46)	-0.06 (-0.99, 0.87)	Unclear
[SOD1], basal	CON (9)	4.5 (-13, 26)	WHEY-CON	-12 (-34, 17)	-0.17 (-0.54, 0.20)	Slight to small
	WHEY (7)	-8.4 (-26, 14)	KDPWHE-CON	-10.6 (-32, 18)	-0.14 (-0.50, 0.21)	Unclear
	KDPWHE (8)	-6.5 (-24, 14)	KDPWHE-WHEY	2.1 (-234, 37)	0.03 (-0.35, 0.41)	Unclear
[SOD1], insulin stimulated	CON (9)	-6.9 (-21, 9.3)	WHEY-CON	21 (-5.3, 55)	0.72 (-0.21, 1.65)	Slight to large
	WHEY (7)	13 (-6.4, 36)	KDPWHE-CON	26 (-0.3, 60)	0.88 (-0.01, 1.78)	Slight to large
	KDPWHE (8)	18 (-1.2, 40)	KDPWHE-WHEY	-12 (-34, 17)	0.16 (-0.81, 1.12)	Unclear
NF-κB, basal^c^	CON (9)	8.9 (-17, 43)	WHEY-CON	-37 (-65, 14)	-0.93 (-2.1, 0.26)	Unclear
	WHEY (10)	-32 (-61, 19)	KDPWHE-CON	-10.9 (-34, 20)	-0.23 (-0.83, 0.37)	Unclear
	KDPWHE (9)	-3.0 (-17, 14)	KDPWHE-WHEY	42 (-20, 148)	0.69 (-0.43, 1.8)	Unclear
NF-κB insulin stimulated^c^	CON (8)	-9.3 (-47, 55)	WHEY-CON	-24 (-57, 34)	-0.72 (-2.2, 0.77)	Unclear
	WHEY (7)	-31 (-50, 4.5)	KDPWHE-CON	10.4 (-37, 93)	0.26 (-1.2, 1.7)	Unclear
	KDPWHE (9)	0.2 (-20, 25)	KDPWHE-WHEY	46 (3.4, 105)	0.98 (0.09, 1.9)	Moderate to large
CS activity, basal	CON (12)	43 (11.4, 82)	WHEY-CON	32 (-3.7, 67)	0.50 (-0.07, 0.93)	Slight to moderate
	WHEY (11)	95 (48, 158)	KDPWHE-CON	-2.2 (-36, 31)	-0.04 (-0.80, 0.49)	Unclear
	KDPWHE (11)	39 (8.2, 80)	KDPWHE-WHEY	-34 (-69, 1.9)	-0.74 (-2.1, 0.03)	Slight to large
COXIV activity, basal	CON (12)	46 (1.2, 19)	WHEY-CON	2.1 (-31, 35)	0.05 (-0.87, 0.71)	Unclear
	WHEY (11)	49 (1.3, 136)	KDPWHE-CON	-19 (-44, 6.6)	-0.49 (-1.4, 0.15)	Slight to large
	KDPWHE (11)	21 (1.2, 1.8)	KDPWHE-WHEY	-21 (-52, 10.5)	-0.55 (-1.8, 0.24)	Unclear
*Erythrocytes*						
[GSH], basal	CON (12)	5.6 (-2.3, 14)	WHEY-CON	-4.0 (-17, 9.4)	-0.23 (-1.1, 0.52)	Unclear
	WHEY (12)	1.6 (-10, 13)	KDPWHE-CON	-3.3 (-17, 10.3)	-0.20 (-1.07, 0.57)	Unclear
	KDPWHE (11)	2.3 (-9.7, 14)	KDPWHE-WHEY	0.6 (-15, 16)	0.04 (-0.94, 0.87)	Unclear
oxiPRX2, basal	CON (12)	0.6 (-1.3, 2.4)	WHEY-CON	1 (-3, 4)	0.20 (-0.76, 1.15)	Unclear
	WHEY (12)	1.3 (-1.7, 4.2)	KDPWHE-CON	-1 (-4, 1)	-0.33 (-1.07, 0.41)	Unclear
	KDPWHE (11)	-0.6 (-2.5, 1.3)	KDPWHE-WHEY	-2 (-5, 2)	-0.49 (-1.52, 0.44)	Unclear

a Refer to Fig. 2 for plots of raw unit point data and distribution statistics, SM Data 1 for detailed statistics, raw measurement units, raw unit point and change-score mean and SD.

b ^-4^Refer Table 2 footer.

c NF-κB DNA binding is of p50 and p65 combined subunits.CON, non-protein isocaloric control; KDPWHE, keratin-derived protein with whey protein blend; WHEY, whey protein isolate. Insulin stimulated is the insulin minus baseline score difference.

Within the skeletal muscle in response to insulin stimulation, after 15-weeks supplementation with WHEY, there was very-good evidence for a large increase in oxiPRX2 vs CON, but effects on oxiPRX3 were unclear (Fig. 2A-B), while the increase in basal oxiPRX2 following KDPWHE vs CON was attenuated with insulin (Fig. 2A, Table 3). Similarly, there was good evidence for a small increases in insulin-stimulated GPx1 (39%) and SOD1 (20%) expression following KDPWHE and WHEY vs CON (Table 3; Fig. 2C-D). By regression analysis we found mechanistic support for a modifying influence of the SOD1-GPx1 dismutase antioxidant pathway on GCR. That is, the mean week 15-0 change of oxiPRX2, GPx1, SOD1 following KDPWHE (Table 3) on GCR was: basal fasted 5% (90%CL -3%, 13%), 11% (-19%, 41%), 1% (-3%, 5%); after insulin-stimulation 4% (0%, 8%), 10% (-24%, 44%), and 1% (-3%, 5%), which adjusted down the primary outcome (KDPWHE-CON on GCR, Table 2) to 19% (-14%, 47%), 1% (-47%, 49%), and 12% (-33%, 57%) for basal; and 17% (-44%, 78%), 4% (-80%, 84%), and 8% (-9%, 25%) for insulin-stimulated, respectively, providing support for a mechanistic association between redox response, endogenous antioxidant expression and glucose clearance, with GPx1 the stronger modifier.

Basal NF-κB (p50/p65) DNA binding was unaffected by either protein supplementation, but there was good evidence for a moderate effect-size increase in insulin-stimulated binding with KDPWHE vs WHEY (Fig. 2E; Table 3). NRF2 concentration was unaffected (Fig. 2F; Table 3). There was good evidence for small (32%) and moderate (34%) increases in basal citrate synthase activity with WHEY compared to CON and KDPWHE, respectively, and good evidence for a small (19%) reduction in cytochrome C oxidase IV activity with KDPWHE vs CON (Table 3).

### 3.5 Insulin signalling, capillarization, and endothelial nitric oxide synthase activity in skeletal muscle

**Fig. 3:**
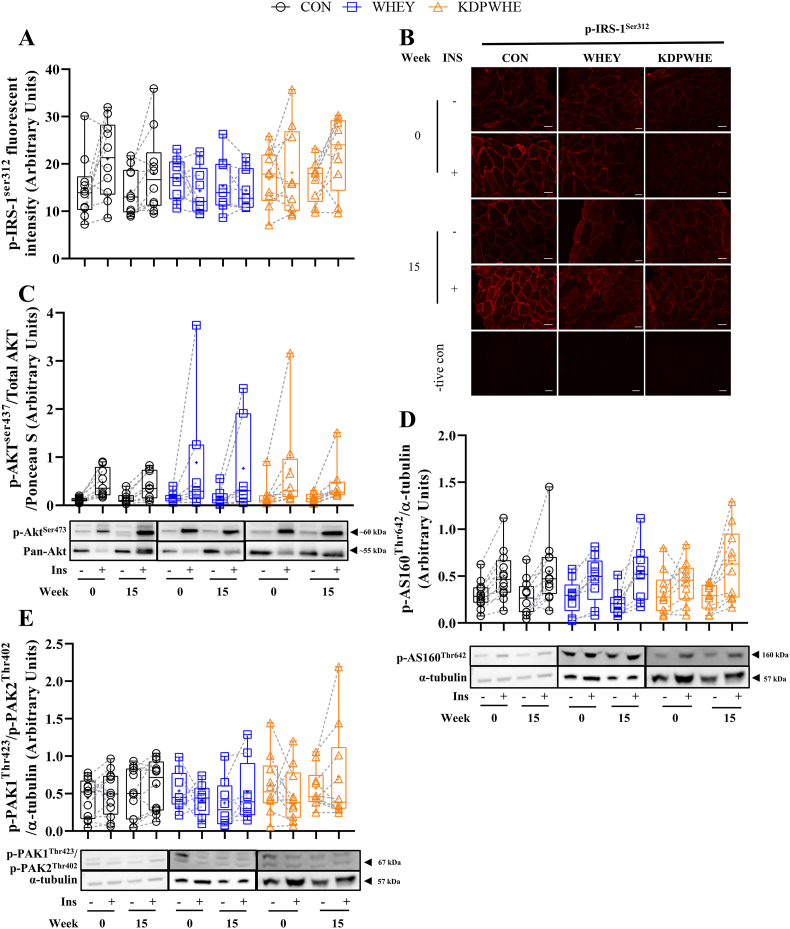
The effect of 14-weeks of CON, WHEY or KDPWHE treatment in adults with type-2 diabetes mellitus on phosphoprotein concentration within the insulin-receptor signalling pathway upstream of regulatory nodes for GLUT4 translocation within the skeletal muscle in responses to basal (INS -) and insulin stimulation (hyperinsulinemic-isoglycaemic; INS +), with data shown as relative protein abundance at week 0 and 15. (A) Insulin-receptor substrate-1 phosphorylated on Ser312 (IRS-1^Ser312^). (B) Representative immunofluorescence images of cross-sectional skeletal muscle fibres stained for IRS-1^Ser312^. IRS-1^ser312^ was measured as staining intensity in the plasma membrane region (SM Fig. 3). The small grey scale lines bottom right represent 50 μm. (C) Protein kinase B phosphorylation on Ser437 (Akt^Ser437^) expressed relative to total Akt. (D) Akt substrate 160 kDa phosphorylation on Thr642 (AS160^Thr642^), (E) p21 activated kinase (PAK) phosphorylation on isoform 1 (PAK^Thr423^) and 2 (PAK^Thr402^). Data are raw unit median, upper and lower quartiles, and range, with individual-participant data points included. Raw unit point and change score mean and SD are in SM Data 1. Outcome statistics are in Table 4. CON, non-protein isocaloric control; KDPWHE, keratin-derived protein with whey; WHEY, whey protein isolate.

Analysis of relative activation of phosphoproteins downstream of the insulin-receptor signalling cascade regulating GLUT4 translocation resolved good evidence for a small (41%) increase (inhibitory action) in insulin-stimulated IRS^Ser312^ phosphorylation, although the KDPWHE vs CON contrast was unclear, and some evidence for a small increase in basal AS160^Thr642^ phosphorylation with KDPWHE vs WHEY (Fig. 3A,B,D, Table 4). Furthermore, there was very-good and strong evidence for moderately lower basal p-PAK1^Thr423^/p-PAK2^Thr402^ phosphorylation with WHEY vs CON (48%) and with WHEY vs KDPWHE (143%), respectively (Fig. 3E; Table 4). WHEY lowered basal Akt^Ser437^/Akt phosphorylation vs CON, but there was no clear effect of KDPWHE. Skeletal muscle capillarization (Capillary count, sharing factor, C/F_i_F ratio and CFPE) increased at week 15, but without any clear effect of supplement (Table 4; SM Fig. 2). NOX2, eNOS and p-eNOS^Ser1177^ were detected in endothelial cells of capillaries and terminal arterioles (SM Fig. 3) as observed previously [35], with some evidence for a slight to small increases with WHEY and with KDPWHE vs CON under basal conditions (Table 4).

**Table 4:** The effect of 14-weeks of KDPWHE, WHEY, or CON treatment in adults with type-2 diabetes mellitus on skeletal muscle tissue insulin signalling, capillarization, and capillary endothelial nitric oxide synthase activity.

Parameter, state	Within-Group Baseline-Adjusted Change^a^		Baseline-Adjusted Treatment-Group Contrast Effect Size Statistics^3^			
	Treatment (n)	Week 15-0 Change in % (90%CL)^b^	Treatment Contrast	Estimate % (90%CL)^b^	Standardized difference (90%CL)^4^	Magnitude
*Insulin signaling phosphoproteins*						
IRS^Ser312^, basal^c^	CON (10)	-6.8 (-23, 13)	WHEY-CON	0.4 (-26, 361)	0.01 (-0.72, 0.74)	Unclear
	WHEY (9)	-6.5 (-26, 19)	KDPWHE-CON	10.2 (-13, 39)	0.23 (-0.33, 0.79)	Unclear
	KDPWHE (9)	2.7 (-11, 18)	KDPWHE-WHEY	9.8 (-17, 45)	0.22 (-0.44, 0.88)	Unclear
IRS^Ser312^, insulin-stimulated^c^	CON (10)	2.6 (-16, 24)	WHEY-CON	-17 (-36, 7.3)	-0.30 (-0.71, 0.11)	Unclear
	WHEY (9)	-15 (-29, 1.4)	KDPWHE-CON	17 (-16, 63)	0.25 (-0.27, 0.77)	Unclear
	KDPWHE (9)	20 (-8.1, 57)	KDPWHE-WHEY	41 (2.5, 95)	0.55 (0.04, 1.06)	Slight to moderate
Akt^Ser437^/Akt, basal	CON (9)	11.1 (-20, 55)	WHEY-CON	-39 (-63, 0)	-1.10 (-2.21, 0.00)	Slight to large
	WHEY(7)	-32 (-53, -2.3)	KDPWHE-CON	-12 (-46, 41)	-0.29 (-1.35, 0.76)	Unclear
	KDPWHE (8)	-2.6 (-31, 37)	KDPWHE-WHEY	44 (-13, 138)	0.81 (-0.30, 1.92)	Unclear
Akt^Ser437^/Akt, insulin-stimulated	CON (9)	-18 (-38, 7.6)	WHEY-CON	35 (-11.3, 106)	0.85 (-0.32, 2.02)	Unclear
	WHEY (7)	10.7, (-23, 59)	KDPWHE-CON	7.1 (-28, 59)	0.14 (-0.84, 1.11)	Unclear
	KDPWHE (8)	-12 (-34, 16)	KDPWHE-WHEY	-21 (-48, 21)	-0.71 (-1.88, 0.46)	Unclear
AS160^Thr642^, basal	CON (12)	7.7 (-87, 808)	WHEY-CON	5.1 (-33, 64)	0.04 (-0.34, 0.42)	Unclear
	WHEY (10)	173 (-16, 789)	KDPWHE-CON	22 (-20, 85)	0.17 (-0.19, 0.53)	Unclear
	KDPWHE (10)	151 (56, 306)	KDPWHE-WHEY	16 (-13, 54)	0.12 (-0.12, 0.37)	Slight to small
AS160^Thr642^, insulin-stimulated	CON (12)	-60 (-92, 99)	WHEY-CON	8.6 (-32, 72)	0.16 (-0.75, 1.07)	Unclear
	WHEY (9)	-43 (-77, 44)	KDPWHE-CON	-10.0 (-39, 347)	-0.20 (-0.96, 0.56)	Unclear
	KDPWHE (10)	-44 (-69, 2.9)	KDPWHE-WHEY	-17 (-43, 21)	-0.36 (-1.09, 0.37)	Unclear
PAK1^Thr423^/						
PAK2^Thr402^, basal	CON (12)	-2 (-62, 152)	WHEY-CON	-48 (-68, -17)	-0.55 (-0.94, -0.16)	Slight to moderate
	WHEY (9)	31 (-50, 241)	KDPWHE-CON	26 (-20, 98)	0.19 (-0.19, 0.57)	Slight to small
	KDPWHE (10)	-71 (-83, -49)	KDPWHE-WHEY	143 (66, 257)	0.74 (0.42, 1.05)	Small to moderate
PAK1^Thr423^/						
PAK2^Thr402^, insulin-stimulated	CON (12)	60 (10.9, 131)	WHEY-CON	22 (-3, 132)	0.20 (-0.42, 0.82)	Unclear
	WHEY (8)	96 (16, 231)	KDPWHE-CON	-15 (-45, 30)	-0.16 (-0.58, 0.26)	Unclear
	KDPWHE (10)	36 (8.5, 70)	KDPWHE-WHEY	-31 (-61, 22)	-0.36 (-0.91, 0.19)	Slight to moderate
*Skeletal muscle capillarization and capillary endothelial nitric oxide synthase enzyme phosphorylation*						
C/F_i_, basal	CON (11)	36 (25, 49)	WHEY-CON	-5.7 (-16.7, 8.7)	-0.21 (-0.64, 0.29)	Unclear
	WHEY (10)	28 (18, 39)	KDPWHE-CON	-4.8 (-18.1, 13.8)	-0.17 (-0.70, 0.45)	Unclear
	KDPWHE (10)	28 (12, 46)	KDPWHE-WHEY	1.1 (-13.6, 21.7)	0.04 (-0.51, 0.69)	Unclear
CFPE, basal	CON (11)	33 (24, 42)	WHEY-CON	7.4 (-9.8, 32.2)	0.37 (-0.54, 1.45)	Unclear
	WHEY (10)	42 (21, 68)	KDPWHE-CON	-12.2 (-24.9, 6.3)	-0.68 (-1.49, 0.32)	Unclear
	KDPWHE (10)	24 (10.6, 39)	KDPWHE-WHEY	-6.6 (-15.2, 3.0)	-0.35 (-0.86, 0.15)	Slight to moderate
eNOS^ser1177^/eNOS, basal	CON (10)	-6.7 (-15, 1.7)	WHEY-CON	11.8 (-8.6, 36.7)	0.21 (-0.17, 0.58)	Slight to small
	WHEY (9)	3.9 (-13, 25)	KDPWHE-CON	12.2 (-4.5, 31.9)	0.21 (-0.09, 0.51)	Slight to small
	KDPWHE (9)	4.4 (-8.8, 19)	KDPWHE-WHEY	0.4 (-19.8, 25.8)	0.01 (-0.41, 0.42)	Unclear
eNOS^ser1177^/eNOS, insulin-stimulated	CON (9)	12 (-4.5, 31)	WHEY-CON	4.8 (-16.6, 31.8)	0.17 (-0.64, 0.97)	Unclear
	WHEY (9)	19 (5.6, 33)	KDPWHE-CON	-4.6 (-26.2, 23.3)	-0.17 (-1.07, 0.74)	Unclear
	KDPWHE (9)	8.6 (-9.7, 31)	KDPWHE-WHEY	-9.0 (-24.8, 10.2)	-0.33 (-1.01, 0.34)	Unclear
^eNOSser1177/NOX2,^ basal	CON (10)	-2.9 (-13, 8.5)	WHEY-CON	6.0 (-8.9, 23.4)	0.16 (-0.26, 0.58)	Slight
	WHEY (9)	3.0 (-7.1, 14)	KDPWHE-CON	8.6 (-10.5, 31.7)	0.23 (-0.30, 0.76)	Unclear
	KDPWHE (8)	5.5 (-9.9, 24)	KDPWHE-WHEY	2.4 (-15.3, 23.8)	0.07 (-0.45, 0.58)	Unclear
^eNOSser1177/NOX2,^ insulin-stimulated	CON (10)	2.0 (-16, 23)	WHEY-CON	7.3 (-13.8, 33.5)	0.29 (-0.60, 1.17)	Unclear
	WHEY (9)	9.5 (-1.8, 22)	KDPWHE-CON	-0.1 (-24.4, 31.9)	0.00 (-1.13, 1.12)	Unclear
	KDPWHE (8)	1.9 (-17, 25)	KDPWHE-WHEY	-6.9 (-26.2, 17.3)	-0.29 (-1.23, 0.65)	Unclear

a Refer to Fig. 3 and SM Figs. 2 and 4, for plots of raw unit point data and distribution statistics, SM Data 1 for detailed statistics, raw measurement units, raw unit point and change-score mean and SD.

b ^-4^Refer Table 2 footer.

c Increased phosphorylation has a negative association with insulin resistance.CON, non-protein isocaloric control; KDPWHE, keratin-derived protein with whey protein blend; WHEY, whey protein isolate; microvascular blood flow (mBF); vasodilation (blood volume, mBV); glucose transporter 4 (GLUT4). Insulin stimulated is the insulin minus baseline score difference.

## 4 Discussion

There was good evidence to suggest that the ingestion of the cysteine-rich keratin-derived combinatorial protein blend (KDPWHE) for 14 weeks by adults with T2DM produced a clinically relevant increase in whole-body glucose clearance rate (GCR), relative to a non-protein placebo control (CON), based on the mean effect size (24%) being comparable to pioglitazone + metformin intervention of similar duration [51]. There was also good evidence for increased GCR with KDPWHE relative to WHEY supplementation, with most insulin-stimulated glucose-delivery mechanisms responses showing no consistent evidence of benefit in response to WHEY relative to CON.

The GCR response following KDPWHE supplementation was supported mechanistically by association with good to very-good level evidence for enhanced nutrient-delivery phenotype and insulin sensitivity within the skeletal muscle – the largest tissue mass responsible for glucose disposal and metabolism [60] – represented by substantially increased mBF and GLUT4 translocation. Insulin-sensitivity within several redox (PRX2; NF-κB) and endogenous antioxidant enzyme protein expression (SOD1, GPx1) responses was also increased by small-moderate mean effect sizes. Consistent with the *a priori* hypothesis, KDPWHE affected muscle redox state associated with GCR, although the response was unexpectedly pro-oxidative at baseline, but pro-antioxidant after insulin exposure. While data from diabetic rodents showed relative hypoglycaemic and oxidative-stress lowering effects of l-cysteine supplementation [61,62] and recent epidemiological analysis suggests reduced cardiometabolic disease mortality with elevated dietary cysteine, glycine and arginine [2], the current empirical data are the first to reveal good evidence for an association between increased dietary intake of these amino acids, tissue redox, and increased insulin sensitivity and blood glucose clearance in humans with T2DM.

The good evidence for improved insulin-stimulated skeletal muscle microvascular blood flow (mBF) and for increased GLUT4 translocation provides for a candidate tissue-level mechanism responsible for the enhanced GCR, because tissue glucose delivery and uptake is partially regulated by insulin-stimulated mBF [20] and by insulin-stimulated GLUT4 translocation [21]. mBF is controlled by NO bioavailability as regulated by eNOS activation [63] influenced by dietary l-arginine availability [9,10]; KDP is relatively high in l-arginine concentration (SM Table 1). Adjusting for mBF eliminated the KDPWHE effect on GCR (adjusted KDPWHE-CON effect -6.4%, 90%CL -62%, 133%), but unfortunately neither l-arginine or NO were measured, and while there was some evidence for a slight-small increase in basal capillary eNOS activity, the insulin-stimulated response was unclear meaning more data is required to confirm or eliminate it as a possible mechanism to explain the increased mBF. Therefore, an explanation for the increased insulin-stimulated mBF with KDPWHE requires further research.

The current data also provide the first indicative evidence to suggest a change in dietary protein pattern can modify insulin-stimulated sarcolemmal GLUT4 density in humans with T2DM. The increase in GLUT4 translocation with KDPWHE supplementation was accompanied with good evidence for a substantially higher IRS^Ser312^ phosphorylation, relative to some evidence for substantial lowering effect with WHEY supplementation, the latter of which may have exerted a negative-feedback inhibitory effect on insulin action [64], but this effect did not translate to any clear evidence for activity on Akt^Ser437^ or AS160^Thr642^ phosphorylation, suggesting insufficient assay resolution or that the insulin-receptor pathway activation was not responsible for increased GLUT4 translocation. Accordingly, good evidence for lower basal p-PAK1^Thr423^/p-PAK2^Thr402^ with WHEY vs KDPWHE suggests changes to RAC1-associated cytoskeletal remodelling may be associated with the relative resistance to exercise-mediated improvement of insulin resistance seen with WHEY vs KDPWHE [65,66]. While we were unable to clearly delineate dietary-protein associated signalling pathways, other clues from rats on a high-fat diet fed cod protein (high arginine, glycine, taurine) show insulin-stimulated (PI)3-kinase/Akt signalling and GLUT4 translocation was preserved, vs soy or casein [67,68]. Additional studies are required to determine the mechanisms by which KDPWHE interacts with redox modifications to increase skeletal-muscle GLUT4 translocation and glucose uptake.

At study onset, we believed that the KDPWHE would increase GSH synthesis, potentially increasing its endogenous antioxidant tissue levels to boost cellular reducing capacity and improve insulin sensitivity, as indicated in prior literature by association [4,6,7]. In contrast, changes in [GSH] in erythrocytes were unclear between KDPWHE compared to CON and WHEY, whereas analytical error precluded inference from our muscle [GSH] and [GSSG] data. One difference would be the mode of ingestion, where Sekhar et al. [7] supplemented with N-acetyl-cysteine, whereas we provided dietary protein. However, peroxiredoxins provide valuable alternative markers of oxidative state [12]. The evidence for elevated basal oxiPRX2 points to a relatively oxidised cytoplasmic redox state in skeletal muscle, which may facilitate the acute response to insulin-stimulated production of reactive oxygen species (ROS), a relevant aspect of the insulin signalling pathway [6,18,[69], [70], [71]]. Accordingly, insulin stimulation was followed by good evidence for increased antioxidant expression and attenuation of the pro-oxidised KDPWHE-CON oxiPRX2 differential. Increased insulin-stimulated GPx1 has been shown to increase the H_2_O_2_ quenching capacity of GSH [72], which could have contributed to moderation of PRX2 oxidation and also dampen the transient cytoplasmic hyper-peroxide response to insulin [[69], [70], [71]], which facilitates glucose transport [73,74]. Meanwhile, increased insulin-stimulated SOD1 expression suggests increased O_2_^•^ − dismutation capacity, which may integrate with the PRX2 and GPx1 responses and counteract the chronic oxidative stress associated with insulin resistance [75,76]. Finally, we noted no clear effect of dietary protein on oxiPRX3, suggesting that the muscle redox-state change is associated with cytosolic events, whereas others have noted a potentially-adaptive compartmentalised mitochondrial redox response in aging muscle [77].

The small-moderate change in basal redox state with KDPWHE may have accumulated over time to drive a comparatively greater adaptive response (hormesis) [78]. ROS are produced in high amounts under hyperlipidaemia or exercise [79], and are thought to enhance metabolic-substrate handling, insulin sensitivity, and mitochondrial adaptation [80,81]. When combined with 4-weeks of exercise in young healthy men, pharmaceutical level supplementation with the exogenous antioxidants vitamins C/E impaired exercise-mediated improvements in insulin sensitivity as measured by glucose infusion rate (GIR) [81]; so at first sight it would seem counter-beneficial to infer that KDP may enhance glucose clearance by facilitating increased tissue reduced endogenous GSH concentration. However, in another sample of young healthy men, the GIR, and also GLUT4, hexokinase II and Akt responses were unaffected with longer-term endurance training [82] suggesting that antioxidant suppression of exercise-mediated adaptations maybe transient. Furthermore, KDP does not contain exogenous antioxidants unlike the high doses of oral antioxidants used by Ristow et al. [81] and the measured thiol redox status (accessible thiol concentration) of the KDP isolate was the same as for whey protein, so the KDP has the same free radical buffering capacity as whey protein isolate alone. Nevertheless, KDPWHE ingestion induced a more oxidised environment within muscle compared to WHEY and CON, and while unmeasured, oxidised cysteine from the ingested KDP could be responsible. Whatever the cause, the more oxidised environment may have been responsible for the improved insulin sensitivity in the current GLUT4 and antioxidant responses. To explore such a metabolic-redox hormesis hypothesis, we analysed the responses of two primary oxidative-stress responders within skeletal muscle: induction of transcription factor NRF2 [83] and NF-κB (p50-p65) transcription factor site binding activity [84,85]. There were no changes in NRF2, but higher relative insulin-stimulated NF-κB DNA binding with KDPWHE may have contributed to increased SOD1 [86] and GPx1 [84] expression. On the other hand, a mixed response to CS and COXIV enzyme activity points to multifactorial regulation, or other unmeasured responses (e.g., S-glutathionylation [87]).

Several limitations should be noted. The study was an exploratory pilot with the motivation to study based by the only prior evidence of any possible glycaemic effect from a mouse trial and from interpolation of a role for redox and tissue glutathione depletion in insulin resistance [7]. Sample size was based on the error for prior GCR responses in non-diabetic individuals and from meaningful minimal effect-size assumptions for change in GCR in response to standard drug interventions. A post-hoc analysis of the current standard error for the GCR outcome for the primary contrast KDPWHE-CON found a sample size of n = 16 per group was required for a 5% rejection error rate, that is, the 90% confidence interval to fall above the threshold (i.e., the SIE 5.4%) for a superior effect size. Increased sample size would have also helped improve statistical precision in skeletal muscle outcomes. In addition, a second limitation associated with sample size was restricted tissue availability from biopsy samples (typically in our samples 120-180 mg, divided into 6 pieces), which for some assays limited sample size. Furthermore, the insulin-stimulated design inherently increased the occurrence of loss of participant values for specific analyses, that is, if only one of the four necessary biopsies contained low or damage tissue sample. One limitation with the biopsy tissue was occasional sampling (approximately one in every 8-10 biopsies) of visible within-sample intermyofibrillar adipose tissue, which required rapid dissection or a second biopsy incision. Additionally, freeze artifact invalidated several samples for immunofluorescence. Readers should be made aware that conducting clinical trials with muscle sampling in people with T2DM is a challenging undertaking. Reliable assay of GSSG was also difficult. In our NEM-treated biopsy samples, we found concentrations of mean 0.13 μm SD 0.30, typically less than 1% (mean 0.7%, SD 1.5%, range 12%–0.2%) of total GSH + GSSG, which was well below the assay technical CV of 4.5%, making the GSH:GSSG ratio as a marker of skeletal-muscle tissue redox state unusable. A more sensitive and reliable mass spectrometry, or other, assay is required for future research on skeletal muscle GSSG concentration and GSH:GSSG ratio, with the clamping of tissue redox state immediate at sampling with NEM essential [12].

Our insulin-sensitivity analysis focus was on traditional and established regulatory processes of relatively macro-level redox markers. However, a myriad of other possible evolutionarily conserved post-translational protein modifications at redox sensitive protein sites may be functional in insulin-signalling or sensitivity processes [6]. Regulatory targets may include alterations in one or more of several different redox forms of cysteine including disulphide bonds, S-glutathionylation, S-nitrosylation, and S-sulfenylation [88]. On the nutritional side, bioavailability studies are required to ascertain the relative digestibility of the amino acids or other bioactive factors within the KDP. We instructed participants to remain on their usual diets throughout the course of the study, but we did not control for changes in background diet, which is both difficult to do in free-living individuals and difficult to measure accurately over 14 weeks; no clear changes in body composition (data not shown) suggested the background diet was unchanged. While the focus of the study was on redox regulation and insulin sensitivity responses in adult men and women with T2DM, the unique amino-acid profile suggests that other conditions such as liver disease [89] and cardiovascular disease [2] may benefit from KDP supplementation. Finally, to avoid bias it should be emphasised that the KDPWHE condition was a blend of KDP and whey protein, suggesting some of the benefits of the treatment might also be attributed to whey protein. However, while similarly affecting some redox measures such as GPx1 and SOD1 expression responses, the current WHEY condition had no clear positive impact on GCR, mBF, or GLUT4 outcomes, and attenuated the improvement in HbA1c in the non-protein placebo control condition, which was partially in line with previous findings [26,90]. One explanation for the difference is that whey protein is rich in branched-chain amino acids, lysine, and methionine, and although complementary with respect to lower levels in KDP, diets rich in these clusters of amino acids have been associated with positive cardiovascular disease mortality risk [2] and T2DM-related metabolic disturbances [91]. Therefore, further research is required to determine any interaction between whey protein and KDP, and to determine if KDP supplementation alone may provide superior cardiometabolic health outcomes.

## 5 Conclusions

The pilot trial provided good evidence to suggest that chronic dietary supplementation with a novel cysteine-rich keratin protein blend may produce a clinically-relevant improvement in whole-body glucose clearance and aspects of muscle insulin sensitivity in humans with T2DM. These changes were associated within good evidence for redox-state shifts responding to insulin, increased antioxidant protein expression, GLUT4 translocation, and higher microvascular blood flow responses within the skeletal muscle tissue. The keratin protein was largely acceptable within baked products and capsules. As cysteine, glycine, and arginine are inversely correlated with cardiovascular disease - the primary cause of death in people with T2DM [2], the current study demonstrates that further research is warranted to verify KDP protein effects on redox and metabolic mechanisms associated with insulin sensitivity with potential as a low-cost dietary supplement for clinical application.

## Funding disclosure

Ministry of Business, Innovation and Employment, NZ [UOOX1404]; Wool Industrial Research Ltd, NZ.

## CRediT authorship contribution statement

Peeters, WM: Conceptualization, Data curation, Investigation, Project administration, Writing - original draft, review & editing, revisions. Gram, M: Conceptualization, Data curation, Formal analysis, Investigation, Methodology, Project administration, Writing - original draft, review & editing. Dias, GJ: Conceptualization, Data curation, Funding acquisition, Methodology, Project administration. Vissers, MCM: Conceptualization, Funding acquisition, Methodology, Project administration, Writing - review & editing. Hampton, MB: Conceptualization, Methodology. Dickerhof, N: Data curation, Investigation, Methodology. Bekhit, AE: Conceptualization, Investigation, Methodology. Black, MJ; Bond, J: Investigation. Oxbøll, J; Bayer, S; Dickens, M; Vitzel, K; Sheard, PW; Perry, BG: Investigation, Methodology. Hodges, LD; Cornwall, J: Conceptualization, Funding acquisition, Project Administration. Brønd, JC: Data curation, Methodology. Stoner, L: Conceptualization, Funding acquisition, Methodology, Writing - review & editing. Rowlands, DS: Conceptualization and overall project management, Research design, Data curation, Statistical analysis, Funding acquisition, Methodology, Project administration, Supervision, Writing – original and final drafts, revisions.

## Declaration of competing interest

We were unable to get the Elsevier online DOI working, so this document covers declarations.

1. George Dias, David Rowlands, and Margreet Vissers received a grant to conduct the study from the New Zealand Ministry of Ministry of Business, Innovation and Employment, NZ [UOOX1404].
2. David Rowlands received additional funding for the project from Wool Industrial Research Ltd, NZ.
3. There is no new patents or other intellectual property files arising from this research.
4. All figures and tables are the authors own and original work.
5. The authors declare not further conflicts of interest.

## Appendix A Supplementary data

SM Fig. 1 CONSORT style summary of participant flow through the trial. SM Fig. 1

SM Fig. 2 Effect of keratin-derived protein on measures of skeletal muscle capillarization. SM Fig. 2

SM Fig. 3 Representative widefield images of skeletal muscle eNOS, p-eNOS^Ser1177^, NOX2, GLUT4 and p-IRS-1^Ser312^ and the effect of keratin-derived protein on insulin. SM Fig. 3
