# Supplementary material for: Changes to insulin sensitivity in glucose clearance systems and redox following dietary supplementation with a novel cysteine-rich protein: A pilot randomized controlled trial in humans with type-2 diabetes
Source: Redox Biol. 2023 Oct 5;67:102918. doi: 10.1016/j.redox.2023.102918 (PMC10570009; doi:10.1016/j.redox.2023.102918)
Supplement: SM Fig. 2 — Effect of keratin-derived protein on measures of skeletal muscle capillarization. [file mmc2.docx]

**Supplemental Figure 1.**

Consort style flow diagram summarizing recruitment and flow of participants through the randomized controlled trial.


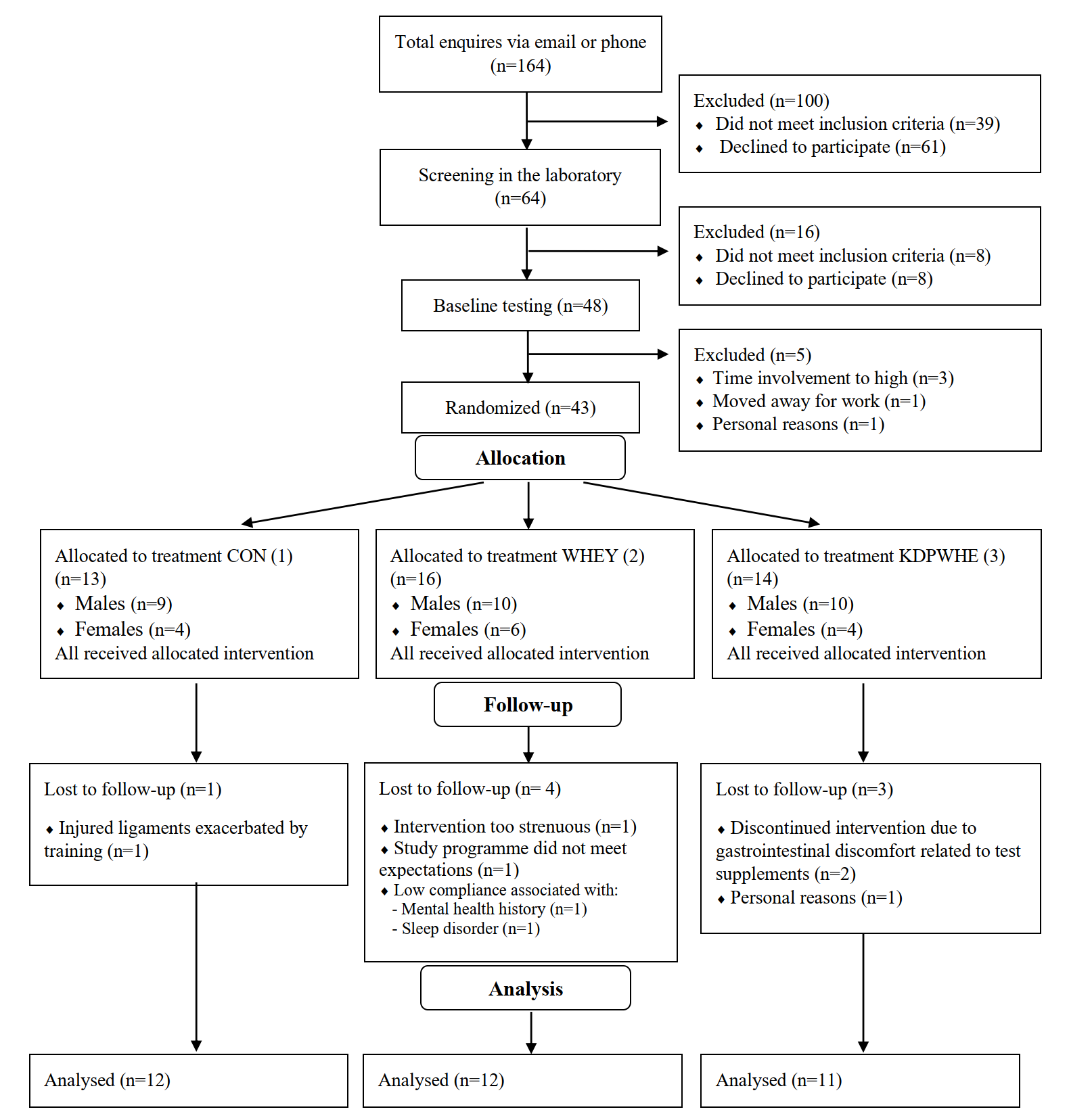


**Supplemental Figure 2.**


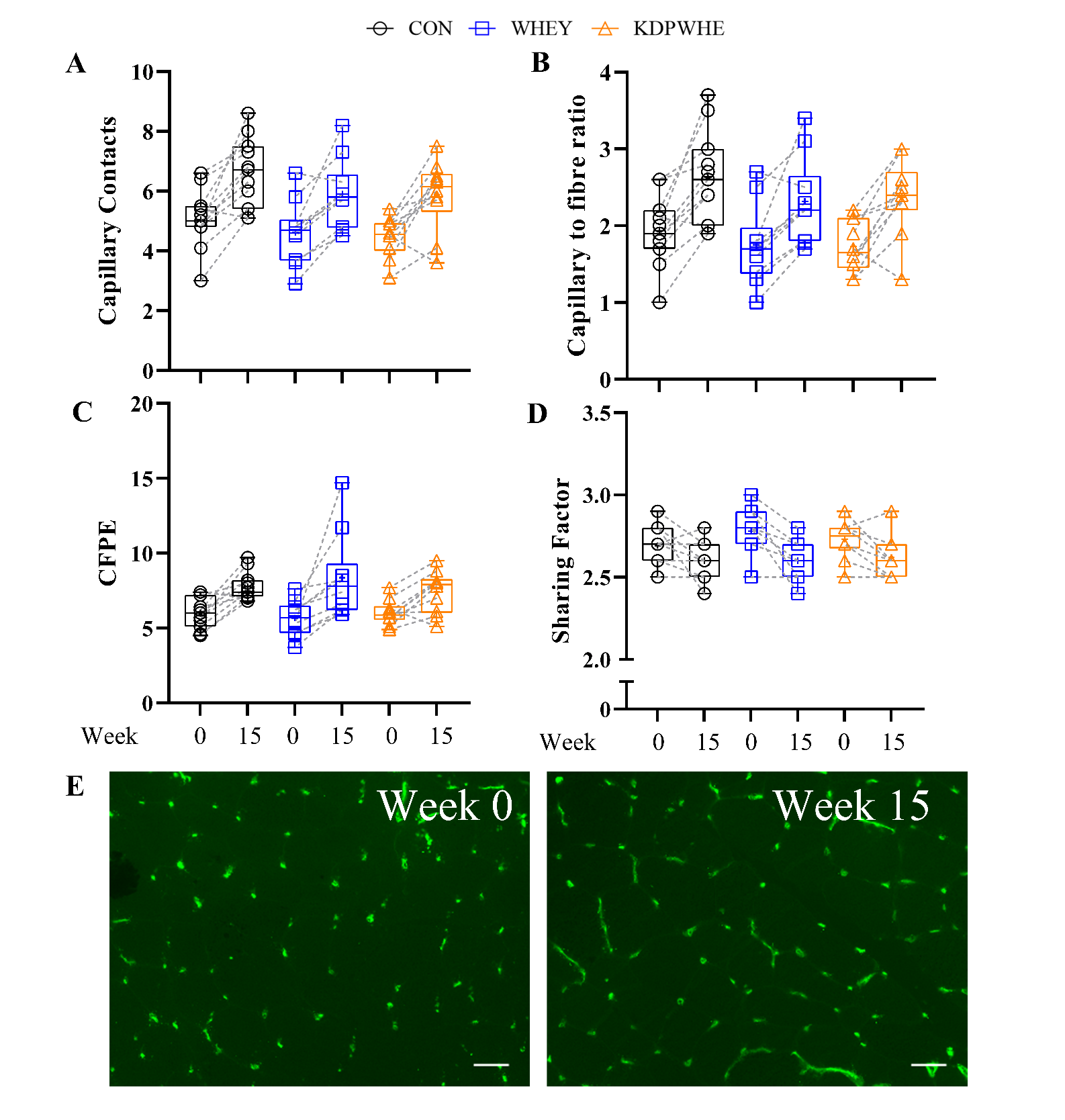


**SM Figure S2.** The effect of 14-weeks of CON, WHEY or KDPWHE treatment in adults with type-2 diabetes mellitus on (A-D) skeletal muscle capillarization parameters before and after dietary intervention and (E) CFPE, capillary-per-fibre perimeter exchange. Capillarization were visualized using Ulex-europaeus-FITC to detect microvascular endothelial cells. Data are median, upper and lower quartiles, range and individual responses. Statistical analysis is in Table 4. Bars are 50 µm. CON, non-protein isocaloric control; KDPWHE, keratin-derived protein with whey; WHEY, whey protein isolate.

**Supplemental Figure 3.**


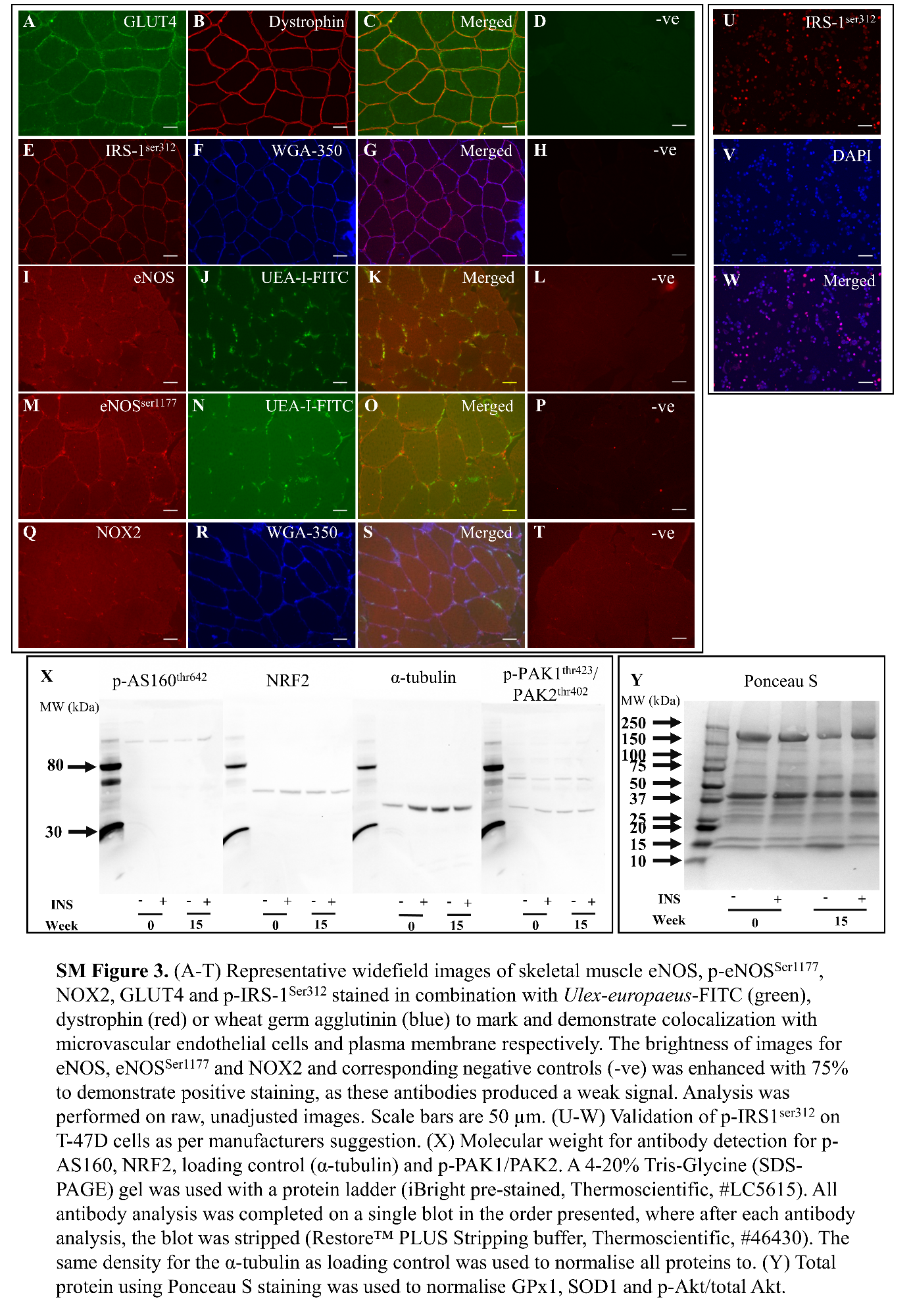


**SM Figure 3.** (A-T) Representative widefield images of skeletal muscle eNOS, p-eNOS^Ser1177^, NOX2, GLUT4 and p-IRS-1^Ser312^ stained in combination with *Ulex-europaeus-*FITC (green), dystrophin (red) or wheat germ agglutinin (blue) to mark and demonstrate colocalization with microvascular endothelial cells and plasma membrane respectively. The brightness of images for eNOS, eNOS^Ser1177^ and NOX2 and corresponding negative controls (-ve) was enhanced with 75% to demonstrate positive staining, as these antibodies produced a weak signal. Analysis was performed on raw, unadjusted images. Scale bars are 50 µm. (U-W) Validation of p-IRS1^ser312^ on T-47D cells as per manufacturers suggestion.

(X) Molecular weight for antibody detection for p-AS160, NRF2, loading control (α-tubulin) and p-PAK1/PAK2. A 4-20% Tris-Glycine (SDS-PAGE) gel was used with a protein ladder (iBright pre-stained, Thermoscientific, #LC5615). All antibody analysis was completed on a single blot in the order presented, where after each antibody analysis, the blot was stripped (Restore™ PLUS Stripping buffer, Thermoscientific, #46430). The same density for the α-tubulin as loading control was used to normalise all proteins to. (Y) Total protein using Ponceau S staining was used to normalise GPx1, SOD1 and p-Akt/total Akt. Statistical outcomes are in Tables 3 and 4.

**Supplemental Figure** **4**.


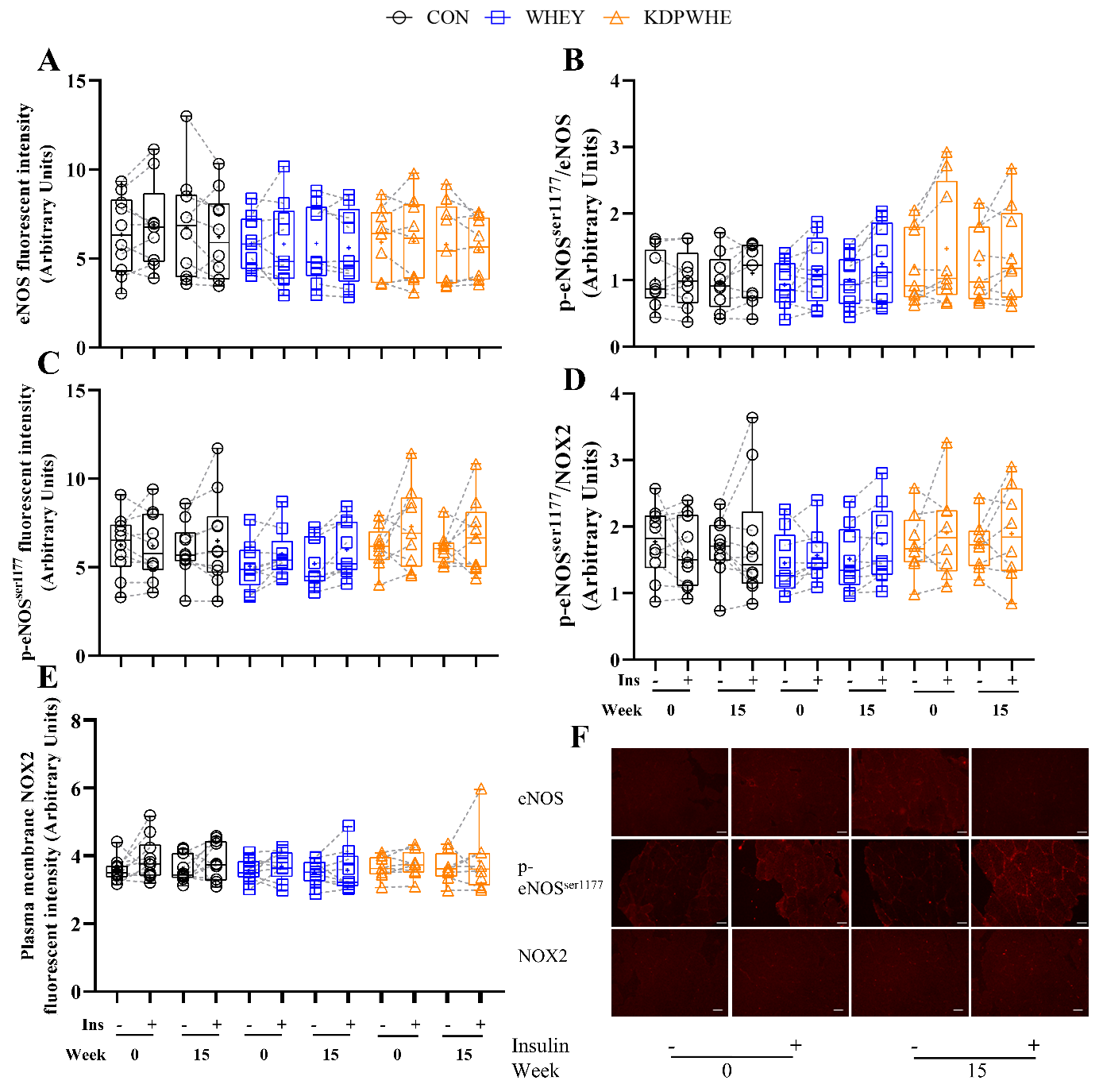


**SM Figure 4**. The expression of microvascular (A) eNOS, (B) p-eNOSS^er1177^/eNOS ratio, (C) p-eNOS^Ser1177^ ratio (D) p-eNOS^ser1177^/NOX2 ratio, and (E) plasma membrane NOX2 before and after 14-weeks of CON, WHEY or KDPWHE treatment in adults with type-2 diabetes mellitus under basal conditions (-) or 1 h into a hyperinsulinaemic-isoglycaemic clamp (+). Immunofluorescence images (F) under basal conditions (-) and 1 h into a hyperinsulinaemic-isoglycaemic clamp (+) before and after the intervention. Bars are 50 µm. The brightness of immunofluorescence images was enhanced by 75% to improve visual presentation. Statistical outcomes are in Table 4. CON, non-protein isocaloric control; KDPWHE, keratin-derived protein with whey; WHEY, whey protein isolate.
