# Supplementary material for: Changes to insulin sensitivity in glucose clearance systems and redox following dietary supplementation with a novel cysteine-rich protein: A pilot randomized controlled trial in humans with type-2 diabetes
Source: Redox Biol. 2023 Oct 5;67:102918. doi: 10.1016/j.redox.2023.102918 (PMC10570009; doi:10.1016/j.redox.2023.102918)
Supplement: SM Fig. 3 — Representative widefield images of skeletal muscle eNOS, p-eNOSSer1177, NOX2, GLUT4 and p-IRS-1Ser312 and the effect of keratin-derived protein on insulin. [file mmc3.docx]

| **Supplemental Table 1.** The macronutrient and amino acid composition of the KDP protein components and the blend used within the trial^1^. | | | | | |
| --- | --- | --- | --- | --- | --- |
|  | Supernatant | Plug | Precipitate | KDP (50/50% plug:precipitate) | WHEY^2^ |
| *Proximate analysis, g/100g* | | | | | |
| Protein | N/A | 86.9 | 86.5 | 86.7 | 93.5 |
| Carbohydrate | N/A | 0.0 | 0.0 | 0.0 | 0.9 |
| Fat | N/A | 0.1 | 0.0 | 0.1 | 0.4 |
| Moisture | N/A | 2.9 | 3.1 | 3.0 | 4.7 |
| Ash | N/A | 2.8 | 2.8 | 2.8 | 1.5 |
| Other | N/A | 7.3 | 7.5 | 7.4 | N/A |
| *Amino acid analysis, g/100g^3^* | | | | | |
| Alanine | 3.0 | 2.9 | 3.0 | 2.9 | 4.8 |
| Arginine | 6.3 | 6.5 | 8.3 | 7.4 | 2.0 |
| Aspartic acid | 4.7 | 1.8 | 2.9 | 2.4 | 9.6 |
| Cysteine | 2.8 | 11.8 | 5.9 | 8.8 | 2.5^4^ |
| Glutamic acid | 10.1 | 8.4 | 12.2 | 10.3 | 16.1 |
| Glycine | 2.6 | 4.2 | 4.1 | 4.2 | 1.6 |
| Histidine | 0.6 | 0.5 | 0.7 | 0.6 | 1.6 |
| Isoleucine | 2.2 | 2.7 | 3.3 | 3.0 | 6.1 |
| Leucine | 5.3 | 5.1 | 7.4 | 6.2 | 9.6 |
| Lysine | 1.9 | 1.8 | 2.5 | 2.2 | 8.7 |
| Methionine | 0.4 | 0.4 | 0.4 | 0.4 | 2.0 |
| Phenylalanine | 1.8 | 2.9 | 3.9 | 3.4 | 2.8 |
| Proline | 3.2 | 7.2 | 4.3 | 5.7 | 5.4 |
| Serine | 4.1 | 6.2 | 5.4 | 5.8 | 4.3 |
| Threonine | 3.2 | 5.2 | 4.3 | 4.8 | 6.3 |
| Tryptophan | 0.5 | 0.7 | 0.8 | 0.7 | 1.6 |
| Tyrosine | 2.5 | 4.3 | 5.4 | 4.8 | 2.9 |
| Valine | 3.7 | 5.3 | 4.8 | 5.1 | 5.4 |
| ^1^Proximate analysis and amino acid composition of the protein powder used for the supplements. KDP, keratin-derived protein; N/A, not available; WHEY, whey protein isolate. Other was unable to be fully defined within the analysis, but probably contains the food acids used in manufacturing (citric and ascorbic acid) and non-protein nitrogen.  ^2^Whey protein isolate (WHEY) used in human trials was from Alacen895, Fonterra, New Zealand.  ^3^The measured amount of amino acids in KDP is 78.7g/100g, the remaining part presumably being selenocysteine and pyrolysine.  ^4^The cysteine content was not available in the whey protein isolate, so the value represents both cystine and cysteine. | | | | | |

| **Supplemental Table 2.** Total daily intake of macronutrients and amino acids for the three different treatments in the form of muffins and capsules. | | | |
| --- | --- | --- | --- |
|  | CON | WHEY | KDPWHE |
| *Macronutrients* | | | |
| Protein, g/day | 3.5 | 42.7 | 42.6 |
| Carbohydrate, g/day | 66.8 | 30.8 | 29.8 |
| Fat, g/day | 26 | 26 | 26 |
| Energy, kJ/day | 1958 | 1978 | 1951 |
| *Amino acid analysis, g/day* | | | |
| Alanine | 0.1 | 2.0 | 1.7 |
| Arginine | 0.1 | 0.9 | 1.8 |
| Aspartic Acid | 0.2 | 4.0 | 2.8 |
| Cysteine | 0.0 | 1.0 | 2.1 |
| Glutamic Acid | 0.3 | 6.8 | 5.8 |
| Glycine | 0.1 | 0.7 | 1.2 |
| Histidine | 0.0 | 0.7 | 0.5 |
| Isoleucine | 0.1 | 2.5 | 2.0 |
| Leucine | 0.1 | 4.0 | 3.4 |
| Lysine | 0.1 | 3.6 | 2.4 |
| Methionine | 0.0 | 0.9 | 0.6 |
| Phenylalanine | 0.1 | 1.2 | 1.3 |
| Proline | 0.1 | 2.3 | 2.3 |
| Serine | 0.1 | 1.8 | 2.1 |
| Threonine | 0.1 | 2.6 | 2.3 |
| Tryptophan | 0.1 | 0.7 | 0.6 |
| Tyrosine | 0.1 | 1.3 | 1.6 |
| Valine | 0.1 | 2.3 | 2.2 |
| Amino acids present in CON are explained by the presence of protein in the supplement ingredients. CON, non-protein isocaloric control; KDPWHE, keratin-derived protein with whey; WHEY, whey protein isolate. | | | |

| **Supplemental Table 3.** List of ingredients and method of preparation used to make the muffins containing the experimental proteins. | | | |
| --- | --- | --- | --- |
|  |  | Treatment |  |
| Ingredients (g) | CON | WHEY | KDPWHE |
| Whey | 0 | 74 | 46 |
| KDP | 0 | 0 | 28 |
| Maltodextrin | 37 | 0 | 0 |
| Gluten free flour | 37 | 0 | 0 |
| Butter | 45 | 45 | 45 |
| Golden syrup | 30 | 45 | 30 |
| Egg | 25 | 25 | 25 |
| Flour | 5 | 20 | 5 |
| Ginger | 4 | 4 | 4 |
| Passion fruit flavour | 1 | 1 | 1 |
| Baking soda | 3 | 3 | 3 |
| Sucralose | 20 mg | 20 mg | 40 mg |
| Guar gum | 20 mg | 20 mg | 20 mg |
| Cinnamon | 1 | 1 | 1 |
| Dates | 30 | 0 | 30 |
| Apricots | 25 | 25 | 25 |
| Coconut thread | 20 | 20 | 20 |
| Vanilla essence | 7 | 7 | 7 |
| Clove oil | 0.7 | 0.7 | 0.7 |
| 0.1M NaOH | 4 | 4 | 4 |
| Rice wine | 40 | 40 | 40 |
| Water | 60 | 60 | 60 |
| Recipe allows for 4 muffins each weighing 70g. Preparation. Chop the ingredients into small pieces and mix the ingredients into a smooth dough. Add 40g of rice wine and 60 ml of water cook for 45 seconds in the microwave. Let the mix rest for an hour before baking. Cook at 150 degrees C for 15 minutes. | | | |

| **Supplemental Table 4.** Mineral composition of the nutritional supplements. | | | |
| --- | --- | --- | --- |
| Minerals | CON | WHEY | KDPWHE |
| Li | 0.048 | 0.056 | 0.062 |
| B | 2.78 | 2.85 | 4.44 |
| Na | 4850 | 5540 | 5960 |
| Mg | 263.5 | 286.5 | 311.5 |
| Al | 8.61 | 11.4 | 12.6 |
| P | 828 | 557 | 537 |
| K | 3525 | 2660 | 3745 |
| Ca | 584 | 970 | 839 |
| V | 0.042 | 0.060 | 0.053 |
| Cr | 0.093 | 0.035 | 0.232 |
| Mn | 5.00 | 6.21 | 5.71 |
| Fe | 14.8 | 18.6 | 17.6 |
| Co | 0.017 | 0.028 | 0.019 |
| Ni | 0.144 | 0.208 | 0.213 |
| Cu | 1.44 | 1.17 | 2.26 |
| Zn | 4.14 | 3.65 | 6.34 |
| Ga | <0.015 | <0.015 | <0.015 |
| As | 0.016 | 0.015 | 0.017 |
| Se | 0.050 | 0.114 | 0.090 |
| Rb | 3.55 | 3.55 | 4.07 |
| Sr | 5.53 | 7.89 | 7.28 |
| Y | 0.007 | 0.008 | 0.007 |
| Mo | 0.053 | 0.081 | 0.088 |
| Ag | <0.0025 | <0.0025 | <0.0025 |
| Cd | 0.007 | 0.006 | 0.007 |
| Sn | <0.05 | <0.05 | <0.05 |
| Sb | 0.015 | 0.015 | 0.011 |
| Cs | 0.018 | 0.020 | 0.023 |
| Ba | 0.645 | 0.844 | 0.807 |
| La | 0.007 | 0.009 | 0.007 |
| Ce | 0.015 | 0.015 | 0.017 |
| Pr | 0.002 | 0.002 | 0.002 |
| Nd | 0.006 | 0.008 | 0.008 |
| Sm | <0.002 | 0.003 | <0.002 |
| Eu | <0.0005 | 0.0006 | <0.0005 |
| Gd | <0.002 | 0.002 | 0.002 |
| Dy | 0.001 | 0.001 | 0.001 |
| Ho | 0.0003 | 0.0003 | 0.0003 |
| Er | 0.001 | 0.001 | 0.0009 |
| Tm | <0.0005 | <0.0005 | <0.0005 |
| Yb | 0.001 | 0.001 | 0.001 |
| Lu | <0.0005 | 0.0006 | <0.0005 |
| Pb | 0.023 | 0.03 | 0.035 |
| Th | 0.008 | 0.008 | 0.009 |
| U | 0.002 | 0.003 | 0.003 |
| Units are mg/kg of total protein. | | | |

| **Supplementary Table 5.** Summary of adverse events during the trial that were likely attributable to the nutritional interventions. | |
| --- | --- |
| Participant | Treatment and symptoms |
| 2 | KDPWHE. Severe GI distress in week 1 (nausea, diarrhoea, not sleeping well, vomiting).  Dropout. |
| 7 | KDPWHE. Severe GI distress in week 1 (severe abdominal pain, bloating, nausea, loss of appetite, urging stools, not sleeping well, vomiting).  Dropout. |
| 9 | KDPWHE. Severe GI distress progressing from half-way through the study (urging stools, diarrhoea, flatulence and abdominal pain). Completed treatment. |
| 11 | WHEY. Lower Respiratory distress (excessive coughing and difficulty breathing). As a result, supplements were blended with moisture (banana, almond milk and water), no further issues.  Completed treatment. |
| 19 | CON. Vomited up capsules. Likely caused by a gastric sleeve.  Dropout. |

**Representative Gastrointestinal Symptoms Questionnaire outcomes**

To illustrate the general response, we present the analysis of the gastrointestinal symptom questionnaire at end of week 1 and 14.

On a linear 0-15 cm scale, participants marked the overall score for any abdominal or epigastric discomfort (cm) at week 1: 0.6 (SD 1.1) in CON, 1.9 (2.4) in WHEY, and 1.9 (2.7) in KDPWHE. By week 14, values were lower at 0.3 (0.5), 0.7 (1.0), and 1.5 (2.7), respectively. Accordingly, the discomfort was higher (1.3 cm; 90%CI 0.2, 2.4) with KDPWHE vs CON during week 1 which disappeared by week 14.

On the 1-7 Likert scale, mean nausea scores were ≤0.5 (effects unclear or trivial). Mean belching scores were ≤0.9, vs CON, belching with KDPWHE was lower (-0.7 scale units; -1.2, -0.2) at week 1, but the same contrast higher by week 14 (0.5; -0.2, 1.2); other differences unclear. Relative to CON neither protein affected flatulence at week 1 (mean score range 0.9 to 1.6), but by week 14 both WHEY (1.1; 0.4, 1.9) and KDPWHE (1.1; 0.4, 1.9) likely increased flatulence. Diarrhoea was unaffected at week 1, but by week 14 there were small increases with both WHEY (0.8; -0.1, 1.6) and KDPWHE (0.7; 0.0, 1.5), vs CON.
